# Differences in muscle energy metabolism and metabolic flexibility between sarcopenic and nonsarcopenic older adults

**DOI:** 10.1002/jcsm.12932

**Published:** 2022-02-17

**Authors:** Marni E. Shoemaker, Suzette L. Pereira, Vikkie A. Mustad, Zachary M. Gillen, Brianna D. McKay, Jose M. Lopez‐Pedrosa, Ricardo Rueda, Joel T. Cramer

**Affiliations:** ^1^ College of Health Sciences The University of Texas at El Paso El Paso TX USA; ^2^ Abbott Nutrition Columbus OH USA; ^3^ Nutrition Science Consulting LLC Galena OH USA; ^4^ Department of Kinesiology Mississippi State University Mississippi State MS USA; ^5^ Department of Health Professions Creighton University School of Medicine Omaha NE USA; ^6^ Abbott Nutrition R&D Granada Spain

**Keywords:** Metabolic flexibility, Sarcopenia, Carbohydrate oxidation, Fat oxidation, Ageing, Metabolism, Exercise

## Abstract

**Background:**

Metabolic flexibility is the ability of skeletal muscle to adapt fuel utilization to the demand for fuel sources [carbohydrates (CHO) and fats (FAT)]. The purpose of this study was to explore muscle energy metabolism and metabolic flexibility under various conditions in sarcopenic (S) versus nonsarcopenic (NS) older adults.

**Methods:**

Twenty‐two older adults aged 65 years or older were categorized as NS [*n* = 11; mean ± standard deviation (SD); age = 73.5 ± 6.0 years (males, *n* = 5; females, *n* = 6)] or S [*n* = 11; 81.2 ± 10.5 years (males, *n* = 6; females, *n* = 5) based on handgrip strength, body composition and physical performance. Indirect calorimetry was recorded before and after consumption of a high‐CHO meal and during aerobic and anaerobic exercise. Respiratory quotient (RQ), CHO and FAT oxidation were assessed. Venous blood samples were collected for glucose and insulin concentrations.

**Results:**

At rest, compared with NS, S exhibited a 5–8% higher RQ at 0 (0.72 vs. 0.76) and 120 (0.77 vs. 0.82), 150 (0.76 vs. 0.80), and 180 min (0.74 vs. 0.80) (*P* = 0.002–0.025); 59–195% higher CHO oxidation at 0, 120, and 180 min (0.0004–0.002 vs. 0.001–0.002 g·min^−1^·kg^‐1)^ (*P* = 0.010–0.047); and 20–31% lower FAT oxidation at 0, 15, and 90–180 min (0.0009–0.0022 vs. 0.0011–0.002 g·min^−1^·kg^−1^) (*P* = 0.004–0.038). Glucose levels were significantly elevated in S versus NS at 0, 60 and 75 min (144.64–202.78 vs. 107.70–134.20 mg·dL^−1^) but not insulin. During aerobic exercise, RQ was 5% greater (0.90 vs. 0.86) (*P* = 0.039), and FAT oxidation was 35% lower at 6–8 min (0.003 vs. 0.005 g·min^−1^·kg^−1^) (*P* = 0.033) in S versus NS. During anaerobic exercise, CHO oxidation was 31% greater in NS versus S at 60–80% time to exhaustion (0.011 vs. 0.007 g·min^−1^·kg^−1^) (*P* = 0.015). Per cent contribution to energy expenditure was greater in S for CHO but lower for FAT at 0 (CHO: 22% vs. 10%; FAT: 78% vs. 91%) and 120–180 min (CHO: 35–42% vs. 17–25%; FAT: 58–65% vs. 75%–84%) (*P* = 0.003–0.046) at rest and 6–8 min during aerobic exercise (CHO: 70% vs. 57%; FAT: 30% vs. 45%) (*P* = 0.046).

**Conclusions:**

The data show differences in skeletal muscle energy metabolism and substrate utilization between S and NS at rest, transitioning from fasted to fed state, and during exercise. Compared with NS, S displayed a diminished ability to adapt fuel utilization in response to feeding and exercise, reflecting metabolic inflexibility. Impaired metabolic flexibility could be a mechanism underlying the losses of strength and physical function accompanying sarcopenia.

## Introduction

Age‐related progressive loss of skeletal muscle mass and strength leading to loss of physical function, a condition called sarcopenia, is associated with increased adverse outcomes including falls, mobility‐disability, frailty, and mortality.^1^ Thus, maintenance of both mass and function of skeletal muscle is critical for healthy ageing.^2^ Although loss of skeletal muscle mass is important, that alone does not fully explain impairments in muscle strength/physical performance.^3^ Although not completely understood, muscle strength and performance are also affected by other age‐related changes affecting its quality/structure (e.g. infiltration of skeletal muscle by fat and connective tissue), neuromuscular function, blood perfusion and delivery of oxygen to tissues, and/or mitochondrial function.

A relatively unexplored area in understanding age‐related declines in muscle strength and performance is muscle energy utilization. To work efficiently, muscles must generate adenosine triphosphate (ATP) needed for muscle fibre contractions. A variety of processes generate ATP, but the most efficient is from the oxidation of fuel sources through glycolysis or beta oxidation of fatty acids.^4^ During the transition from fasted to fed states or vice versa, energy substrate availability to skeletal muscles changes; thus, skeletal muscle metabolism must be able to adapt to utilize the available fuel source (lipid and/or carbohydrate).

The lucid ability of skeletal muscle to adjust its metabolism is a healthy state and has been termed metabolic flexibility.^5^, ^6^ Likewise, during the transition from rest to exercise, energy demands of skeletal muscle increase dramatically and requires a similar ability to adjust immediately to primarily carbohydrate (CHO) (to a lesser extent, lipid) availability.^5^, ^6^ An inability to quickly adjust and utilize available energy substrates has incidentally been termed metabolic ‘inflexibility’.^6^ Metabolic inflexibility has been associated with insulin‐resistant conditions such as obesity and type 2 diabetes, which are also linked to impaired muscle performance.^5^, ^6^ However, still unknown is the metabolic flexibility status of people with sarcopenia.

The importance of fuel availability to support muscle performance and reduce fatigue during endurance activities is well established in the field of sports nutrition.^7^ Less is known about how the ability to utilize these energy substrates could impacts muscle endurance/fatigue in older men and women. Numerous studies comparing older versus younger adults shows that older individuals have reduced muscle glucose uptake, reduced glucose transporter (GLUT 4) expression, greater insulin resistance, reduced muscle mass, increased intramyocellular fat content and reductions in rates of muscle mitochondrial activity.^8^, ^9^, ^10^, ^11^ Individually and collectively, these differences would be expected to impact uptake and oxidation of energy substrates to generate ATP needed for muscle performance.^6^ However, while the ability to utilize energy substrates may be less efficient during ageing,^12^ how this relates to muscle performance is unknown. In addition to facilitating whole‐body locomotor function, skeletal muscle represents the body's largest metabolically active protein reservoir and glucose disposal site. In fact, skeletal muscles are responsible for approximately 75% of whole‐body insulin‐stimulated glucose uptake.^2^ Given these crucial functional and metabolic duties ascribed to muscle, it is important to better understand muscle energy metabolism and metabolic flexibility under various physiological states in sarcopenic (S) versus nonsarcopenic (NS) older male and female adults.

## Methods

### Study design

**Figure 1:**
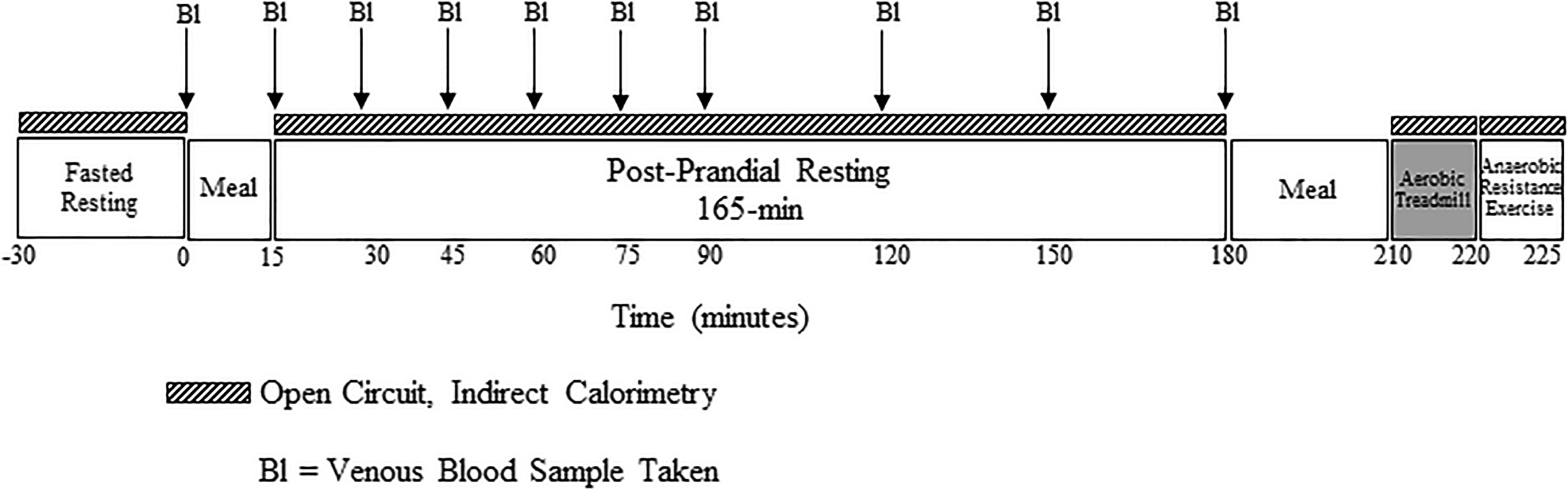
Test visit activity timeline. Resting metabolism was measured for 30 min prior to and 180 min after consuming a CHO‐rich meal. Venous blood samples (Bl) were taken fasted before the meal (baseline) and every 15 ± 5 min for the first 90 min, and then approximately every 30 ± 5 min up to 180 min.

A single‐centre, cross‐sectional design was used. Participants underwent prescreening, and if qualified, were invited to participate in the screening visit. If the participant qualified after the screening visit, a test visit in the laboratory was scheduled 4 to 10 days after the screening visit. The prescreening visit was performed by the investigators via telephone, during an in‐home visit or at a public gathering to assess participant eligibility. Potentially eligible participants then came to the laboratory for the full screening visit. During the test visit, resting metabolism and postprandial CHO and fat (FAT) oxidation was monitored. Venous blood was sampled at regular intervals and analysed for glucose and insulin. Figure 1 displays the timeline of the test visit.

### Participants

**Figure 2:**
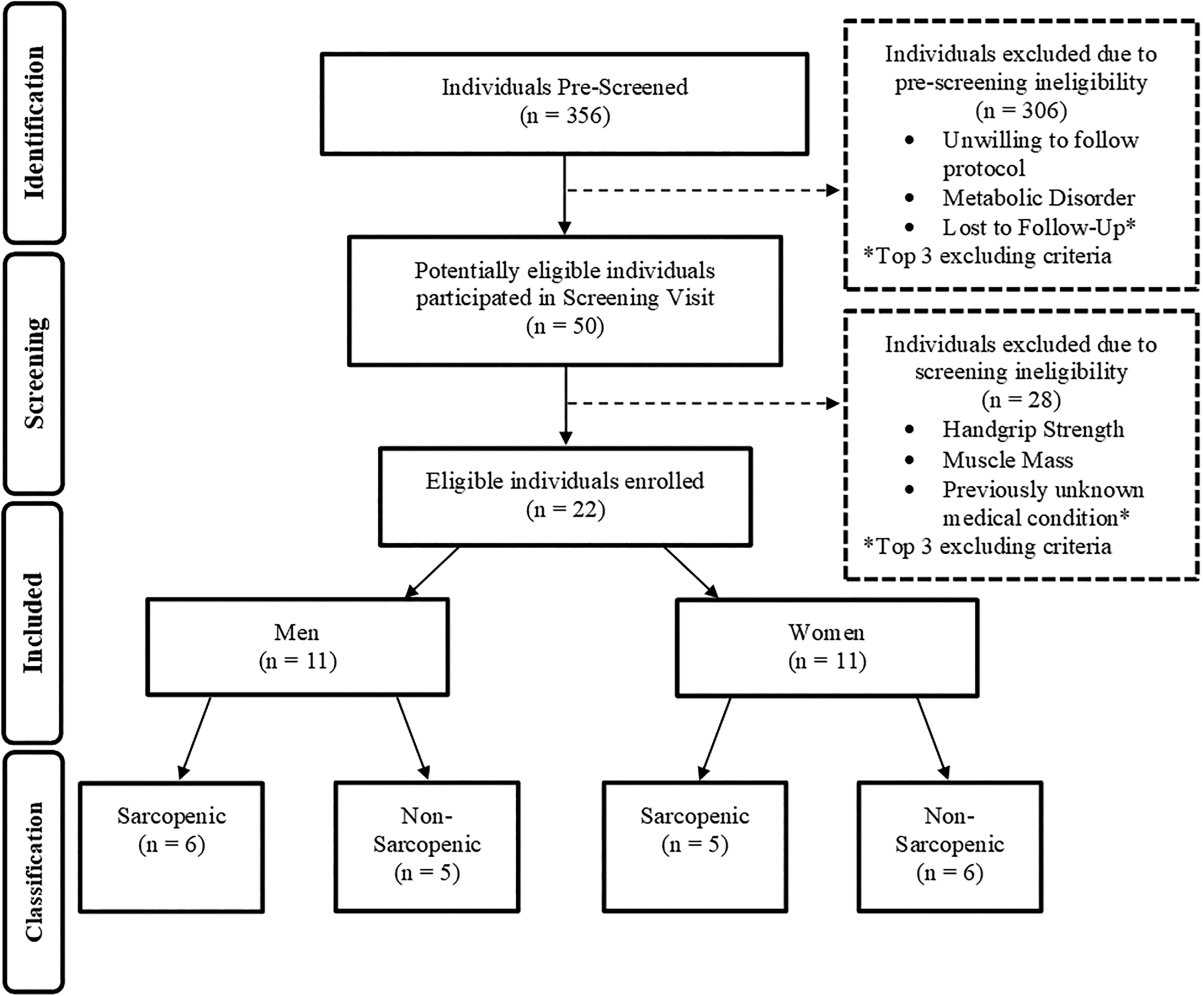
Participant screening and enrolment process overview.

Participants were recruited from previous study databases, community organizations and retirement living communities. Three hundred and fifty‐six individuals were prescreened for eligibility. A total of 22 participants were eligible and enrolled into the study (Figure 2). Participants were included if they met the inclusion and exclusion criteria during the prescreening and screening visit (Table S1).

Participants were included in the study if they met the following inclusion criteria during the prescreening and screening visit: (1) subject is 65 years of age or older at the time of screening; (2) subject's body mass index is ≥18.0 and ≤39.0 kg·m^−2^; (3) subject is ambulatory (able to walk without assistance); (4) subject is not a current smoker (within past 10 years); (5) subject is classified as low OR moderate risk as defined by the American College of Sports Medicine (ACSM) Guidelines for Exercise Testing & Prescription^13^ based on the responses from AHA/ACSM Health/Fitness Facility Preparticipation Screening Questionnaire. The four‐step procedure was (i) ask each participant about risk factors using the AHA/ACSM Health/Fitness Facility Preparticipation Screening Questionnaire,^13^ (ii) count the number of risk factors present using the definitions provided by the ACSM Guidelines^1^ per patient reported history, (iii) classify each participant as low, moderate, or high risk using the classification chart from the ACSM Guidelines,^13^ and (iv) Participant is only included if qualified as low or moderate risk; (6) participants has normal muscle mass and strength/performance (normal grip strength [≥30.0 kg (men); ≥20.0 kg (women)]) to be determined as nonsarcopenic OR low muscle mass and strength/performance (low grip strength [<30 kg (men); <20 kg (women)]) to be determined as sarcopenic, as defined by the European Working Group on Sarcopenia in Older People^14^; (7) if subject is on thyroid medication or hormone replacement therapy, subject states he/she has been on a constant dosage for at least 2 months prior to screening visit; (8) subject states he/she is willing to follow protocol as described; and (9) subject has voluntarily signed the Informed Consent Form.

Participants were excluded from the study if they met any of the following criteria during prescreening, screening visit or test visit: (1) subject states he/she has a history of metabolic/endocrine (diabetes), hepatic, or renal disease, myocardial infarction, peripheral vascular disease, respiratory or neuromuscular disease; (2) subject states he/she regularly participates in a resistance exercise programme; (3) subject states he/she has had poor appetite with recent unexplained weight loss [e.g. 10 pounds (4.5 kg)] over the past 6 months; (4) subject states he/she has a current infection (requiring medication or which might be expected to require hospitalization), has had inpatient surgery, or corticosteroid treatment (excluding topical creams) in the last 3 months or antibiotics in the last 3 weeks prior to the screening visit; (5) subject states that he/she has an active malignancy, excluding carcinoma in situ of the cervix, cutaneous malignancies (basal cell carcinoma, squamous cell carcinoma, except melanoma); (6) subject states that he/she has a chronic, contagious, infectious disease, such as active tuberculosis, hepatitis A, B, or C, or HIV; (7) subject reports currently taking medications/dietary supplements or substances that could profoundly modulate metabolism in the opinion of the principal investigator (PI) or study physician, for example, progestational agents, steroids, growth hormone, dronabinol, marijuana, CaHMB, free amino acid supplements and dietary supplements to aid weight loss or gain. Exceptions included use of multivitamin/mineral supplement, topical or optical steroids and short‐term use (less than 2 weeks) of dexamethasone; (8) subject is known to be allergic or intolerant to any foods; (9) subject states he/she has had history of gastrointestinal disease (e.g. Crohn's, colitis and celiac), or surgeries (including gastric balloon), gastroparesis, or taking medications that are known in the opinion of the PI or study physician (e.g. cholinergic agonists, prokinetic agents, opioid antagonists, antidiarrheals and antibiotics) to interfere with consumption/digestion/absorption of nutrients; (10) subject states he/she has an eating disorder, severe dementia or delirium, history of significant neurological or psychiatric disorder, alcoholism, substance abuse, or other conditions that may interfere with compliance with study protocol procedures in the opinion of the PI or study physician; (11) subject states that he/she is a participant in a concomitant trial or trial of a nonregistered drug (or is within the 30‐day follow‐up period for such a trial).

Prior to participation, all individuals reviewed, signed and dated an Informed Consent Form approved by the University of Nebraska‐Lincoln Institutional Review Board (IRB) for the protection of human subjects (IRB Project # 18543; IRB Approval Date: 6 September 2018). A signed and dated copy was provided to the participant prior to any study participation. This study was registered in clinicaltrials.gov (NCT03701867).

### Screening visit

At the screening visit, demographic and medical information were collected to ensure the participant was eligible based on inclusion and exclusion criteria. Measurements of anthropometrics, handgrip strength (kg), the short physical performance battery (SPPB) and body composition via dual‐energy X‐ray absorptiometry (DXA) were obtained. An estimation of V̇O_2_ and maximal leg extension strength was determined.

### Strength and function assessments

Handgrip strength was measured with a handheld dynamometer (Jamar® Hydraulic Hand Dynamometer, Patterson Medical, Warrenville, IL).^3^ The SPPB^15^ evaluated balance, gait speed at 4 m and endurance. A total score of all three tests was calculated, ranging from 0 to 12, where 0 is the lowest and 12 is the highest level of functionality. Descriptions of these strength and function tests are presented in Table S2.

### Anthropometrics and body composition

Anthropometrics and body composition assessments are described in detail in Data S3. Whole‐body DXA (Lunar iDXA, GE Healthcare, Madison, WI) was used to categorized participants as ‘normal’ or ‘low’ skeletal muscle mass based on relative skeletal muscle index (RSMI) from Kim *et al*.^16^ and Janssen *et al*.^17^ RSMI can be accurately predicted from models using DXA measurements of appendicular lean skeletal tissue (ALST) to determine total body skeletal muscle (TBSM) using the following equations^16^:

$$\text{TBSM}\;\left(\mathrm{kg}\right)=1.13\times \text{ALST})-\left(0.02\times \mathrm{Age}\right)+\left(0.61\times \mathrm{sex}\right)+0.97$$

$$\text{RSMI}=\left(\text{TBSM}\;÷\;\text{Weight}\right)\times 100$$

where ALST = sum of lean tissue in the arms and legs, and sex is coded as 0 = female, 1 = male. Criteria for low RSMI was ≤37% and ≤28% for male and female adults, respectively.^17^

### V̇O_2_max estimation

A nonexercise estimation of V̇O_2_max was performed using a validated equation (*R* = 0.81, standard error of estimate = 4.80 mL·kg^−1^·min^−1^)^18^:

$$\dot{V}O2\max=59.416–\left(0.327\times \mathrm{age}\;\left(\text{years}\right)\right)+\left(11.488\times \mathrm{sex}\right)+\left(1.297\times \text{PASS}\right)-\left(0.266\times \text{waist girth}\;\left(\mathrm{cm}\right)\right)$$

where the physical activity status scale (PASS) represents a score ranging from 0 to 10 determined from the NASA PASS.^19^ Sex was dichotomized as male = 1 and female = 0. This regression model was utilized because it was suggested to be most accurate for older adults aged > 50 years and less active individuals (PASS < 6.5, with the highest prediction accuracy for those over age 50.^18^ The estimation of V̇O_2_max was used to calculate 50–60% of V̇O_2_max.

### Leg extension strength

Maximal leg extension strength was estimated submaximally with a unilateral, dynamic constant external resistance five repetition maximum (RM) test.^20^ Further description of the leg extension assessment is provided in Data S4. Once the 5RM was determined, the external resistance added was recorded and used to estimate the 1RM using the following validated equation (*R* = 0.994, standard error of estimate = 13.51 kg)^20^:

$$1\mathrm{RM}=1.0970\times \left(5\mathrm{RM}\;\text{loaded resistance}\;\left(\mathrm{kg}\right)\right)+14.2546$$

The model using 5RM to predict 1RM strength was more accurate than using a 10 and 20RM prediction equation,^20^ and utilization of a multiple RM was a conservative approach to assessing muscle strength in higher risk patients such as older adults.^13^

### Test visit

Four to ten days after the Screening Visit, eligible participants arrived at the laboratory in the morning after an 8–16‐hour fast with a completed 3‐day dietary recall. Participants were then provided a brief review of the Test Visit procedures.

### Dietary food recall

Participants were instructed to complete a dietary food recall for the 3 days prior to the scheduled test visit. A registered dietitian explained how to fill out the form as completely as possible. Participants were instructed to consume a minimum of 150 g of CHO per day for the 3 days prior to the test visit. This was confirmed visually at each test visit by a registered dietitian and later analysed in a software programme (MyFitnessPal, Under Armour, Inc., 2005). Mean ± standard deviations (SD) of energy intake (kcal·day^−1^) and macronutrient intake (g·day^−1^) were analysed for each day (Table S5).

### Indirect calorimetry and metabolic measurements

Resting metabolism was measured for 30 min prior to and 180 min post‐consumption of a standard high carbohydrate meal that included an English muffin, peanut butter and Gatorade®, with a composition of 51 g of CHO, 9 g of fat and 6 g of protein (Figure 1). Additional descriptions of the metabolic measurements are provided in Data S6. Metabolic measurements [V̇O_2_, V̇CO_2_ and respiratory quotient (RQ)] were exported for signal processing (LabVIEW v. 18.0, National Instruments, Austin, TX). Metabolic measurements were averaged over 10‐min windows with the least variability within the 15 or 30‐min timepoints over the resting period. CHO oxidation rate (g·min^−1^) and FAT oxidation rate (g·min^−1^) were calculated at rest and during aerobic exercise using standard stoichiometric equations^21^:

$$\mathrm{CHO}\;\text{Oxidation Rate}\;\left(g\cdot \min^{-1}\right)=4.55\;\left(\overset{\cdot }{V}\mathrm{CO}2\right)–3.21\;\left(\overset{\cdot }{V}O2\right)–2.87\;\left(n\right)$$

$$\mathrm{Fat}\;\text{Oxidation Rate}\;\left(g\cdot \min^{-1}\right)=1.67\;\left(\overset{\cdot }{V}O2\right)–1.67\;\left(\overset{\cdot }{V}\mathrm{CO}2\right)–1.92\;\left(n\right)$$

CHO and FAT oxidation rate (g·min^−1^) were calculated during anaerobic exercise using a proposed equation for substrate utilization during high‐intensity exercise^21^:

$$\mathrm{CHO}\;\text{Oxidation Rate}\;\left(g\cdot \min^{-1}\right)=4.210\;\left(\overset{\cdot }{V}\mathrm{CO}2\right)–2.962\;\left(\overset{\cdot }{V}O2\right)–2.37\;\left(n\right)$$

$$\mathrm{Fat}\;\text{Oxidation Rate}\;\left(g\cdot \min^{-1}\right)=1.695\;\left(\overset{\cdot }{V}O2\right)–1.701\;\left(\overset{\cdot }{V}\mathrm{CO}2\right)–1.77\;\left(n\right)$$

where *n* represents urinary nitrogen excretion. In the above calculations, CHO and FAT oxidation are based on a V̇O_2_ (STPD) of 2.500 L·min^−1^ and a V̇CO_2_ (STPD) of 2.250 L·min^−1^ and negligible protein oxidation (*n* = 0).^21^ For analysis, CHO and FAT oxidation rates were normalized to kilogram of fat‐free mass (FFM).

### Venous blood samples

Venous blood samples were collected for assessment of glucose and insulin at 10 timepoints during rest. A fasting blood sample was collected at baseline (time ‘0’) and postprandial every 15 ± 5 min for the first 90 min, and then approximately every 30 ± 5 min up to 180 min (Figure 1). Homeostatic model assessment of insulin resistance (HOMA‐IR) was calculated using fasting glucose and insulin concentrations with the following formula derived from Matthews *et al*.^22^:

$$\text{HOMA‐}\mathrm{IR}=\frac{\left[\text{fasting glucose}\;\left(\text{mmol}\cdot L^{-1}\right)\right]\times \left[\text{fasting insulin}\;\left(\mathrm{mU}\cdot L^{-1}\right)\right]}{22.5}$$

Whole‐body insulin sensitivity from the meal tolerance test was calculated using the composite insulin sensitivity index following the formula derived from Matsuda et al.^23^:

$$\frac{10,000}{\sqrt{\mathrm{FG}\times \mathrm{FI})\times \left(\text{Mean OGTT glucose concentration}\times \text{Mean OGTT insulin concentration}\right)}}$$

where FG represents fasting glucose, FI represents fasting insulin and mean OGTT concentrations represents the area under the curve calculated postprandially for glucose and insulin. Further description of blood sample collection and analysis are in Data S7.

### Submaximal aerobic exercise

Prior to the aerobic exercise protocol, participants were provided a second high‐CHO meal of 50 g of CHO, 9 g of fat and 6 g of protein (white bread, cheese and Gatorade®). Indirect calorimetry was measured during a submaximal aerobic exercise protocol based on previous methods.^5^ Participants began the protocol by walking at 2 mph at 0% grade on a treadmill (Vision Fitness; Cottage Grove, WI, USA). After allowing for a 2‐ to 3‐min cardiac adjustment, an evaluation for target speed was made by comparing the current V̇O_2_ to the predetermined estimated V̇O_2_ max. Participants completed a 10‐min walk (0% grade) at a steady‐state, between 50% and 60% of their estimated V̇O_2_ max. Metabolic measurements were exported and averaged over every 2‐min period.

### Anaerobic fatiguing exercise

Participants were secured to the Biodex (Biodex Medical Systems, Inc., Shirley, NY, USA) chair for the anaerobic fatiguing exercise protocol based on previous methods.^24^ The leg extension device (Hammer Strength Plate‐Loaded, Iso‐Lateral Leg Extension Machine; LifeFitness, Rosemont, IL, USA) was loaded with approximately 30% of the estimated 1RM from the screening visit. A metronome was set at a tempo of 20 b.p.m. so that one repetition was completed every 3 s. Participants completed a repetition every time they heard the metronome sound until they were unable to complete another repetition throughout their full range of motion. Number of repetitions and time to exhaustion were recorded. Indirect calorimetry was measured throughout the fatiguing bout. Metabolic measurements were exported and divided into quintiles (%) based on number of repetitions and time to exhaustion and categorized as 0–20%, 20–40%, 40–60%, 60–80% and 80–100% time to exhaustion.

### Statistical analyses

Means, SD and 95% confidence intervals were calculated (Microsoft Excel, Version 1610). Two‐way analyses of variance [sex × sarcopenic status (SS)] compared baseline demographics, anthropometrics, body composition, strength, diet composition and repetitions to failure. Separate three‐way mixed factorial analyses of variance (time × sex × SS) analysed RQ, CHO and FAT oxidation normalized to FFM, per cent substrate contributions to energy expenditure, and concentrations of glucose and insulin. Planned comparisons between NS and S groups were determined a priori and performed with independent samples *t*‐tests. Pearson product correlation coefficients were calculated to examine the relationships between RSMI and handgrip strength with RQ, CHO and FAT oxidation normalized to FFM for the entire sample and males and females separately. All statistical analyses were performed with IBM SPSS v. 25 (Chicago, IL, USA). An alpha of *P* ≤ 0.05 was considered statistically significant for all comparisons.

## Results

Five male participants (mean ± SD; age = 71.8 ± 5.7 years; stature = 172.2 ± 6.0 cm; body mass = 80.2 ± 9.5 kg) and six female participants (age = 75.0 ± 6.4 years; stature = 160.6 ± 7.8 cm; body mass = 62.2 ± 7.2 kg) were categorized as NS. Six male participants (age = 87.0 ± 9.4 years; stature = 167.1 ± 4.3 cm; body mass = 69.0 ± 8.9 kg) and five female participants (age = 74.2 ± 7.3 years; stature = 157.8 ± 8.5 cm; body mass = 71.5 ± 6.6 kg) were categorized as S.

Baseline characteristics (means ± SD) of the 22 participants separated by SS and sex are displayed in Table 1. There were sex × SS interactions for age, body mass index, FFM, ALST, leg strength and repetitions to exhaustion. Compared with the NS group, S individuals were significantly older, had greater BF%, lower RSMI, FFM, and ALST, lower grip strength, slower gait speed, lower SPPB scores, estimated V̇O_2_max, leg extension strength and repetitions to exhaustion. Although there were no statistical differences between NS and S groups for HOMA‐IR, whole‐body insulin sensitivity was 31% lower in S than NS (composite insulin sensitivity index; NS: 0.11 ± 0.07; S: 0.07 ± 0.02; *P* = 0.085). Three‐day dietary records indicated no statistical differences in energy intake or macronutrient intake between groups with the exception of higher CHO intake in male versus female individuals on Day 3 (*P* = 0.046) (Table S5).

**Table 1:** Means ± standard deviations (SD) for baseline demographics, anthropometrics, body composition, strength and repetitions to failure for the nonsarcopenic (*n* = 11) and sarcopenic (*n* = 11) older adults

		Nonsarcopenic		Sarcopenic				
		Males	Females	Males	Females			
Sample size	(*n*)	5	6	6	5	Interaction effect (sarcopenia × sex)	Main effect (sarcopenia	Main effect (sex)
Age	(years)	71.8 ± 5.7	75.0 ± 6.4	87.0 ± 9.4	74.2 ± 7.3	***P* = 0.022**	***P* = 0.036**	*P* = 0.149
Body mass index (BMI)	(kg·m^−2^)	27.0 ± 1.4	24.2 ± 2.2	24.7 ± 3.1	28.8 ± 3.5	***P* = 0.008**	*P* = 0.319	*P* = 0.562
Per cent body fat	(%)	22.6 ± 3.5	36.7 ± 3.0	26.8 ± 6.5	41.8 ± 1.6	*P* = 0.788	***P* = 0.018**	***P* < 0.001**
Fat‐free mass (FFM)	(kg)	58.4 ± 6.9	37.3 ± 5.2	46.6 ± 3.7	38.2 ± 3.3	***P* = 0.007**	***P* = 0.019**	***P* < 0.001**
Appendicular lean soft tissue (ALST)	(kg)	28.3 ± 3.9	16.6 ± 2.1	20.9 ± 2.2	16.4 ± 1.4	***P* = 0.004**	***P* = 0.003**	***P* < 0.001**
Relative skeletal muscle index (RSMI)	(%)	40.1 ± 2.2	29.3 ± 0.9	34.7 ± 2.4	25.9 ± 0.7	*P* = 0.177	***P* < 0.001**	***P* < 0.001**
Handgrip strength	(kg)	42.1 ± 8.5	25.1 ± 5.2	27.7 ± 2.1	16.5 ± 2.4	*P* = 0.204	***P* < 0.001**	***P* < 0.001**
Gait speed	(m·s^−1^)	1.2 ± 0.1	1.1 ± 0.2	1.0 ± 0.2	0.9 ± 0.2	*P* = 0.533	***P* = 0.008**	*P* = 0.210
Short physical performance battery (SPPB)	(score)	12.0 ± 0.0	11.5 ± 0.8	10.5 ± 1.0	10.2 ± 1.6	*P* = 0.826	***P* = 0.006**	*P* = 0.385
Estimated V˙O_2_max	(mL·kg^−1^·min^−1^)	32.1 ± 4.9	22.2 ± 2.9	26.6 ± 4.2	17.9 ± 5.4	*P* = 0.731	***P* = 0.017**	***P* < 0.001**
Leg extension strength (5RM)	(kg)	19.5 ± 1.3	9.5 ± 2.5	11.7 ± 3.3	7.5 ± 1.9	***P* = 0.013**	***P* < 0.001**	***P* < 0.001**
Repetitions to exhaustion @ 30% 1RM	(reps)	26.6 ± 10.1	13.2 ± 2.9	14.5 ± 3.6	13.4 ± 6.2	***P* = 0.030**	***P* = 0.036**	***P* = 0.012**
HOMA‐IR	(molar units)	1.1 ± 0.6	0.7 ± 0.4	1.0 ± 0.2	1.4 ± 0.6	*P* = 0.100	*P* = 0.162	*P* = 0.982

*P* values are type I errors of the interactions and main effects.

### Resting metabolism

**Figure 3:**
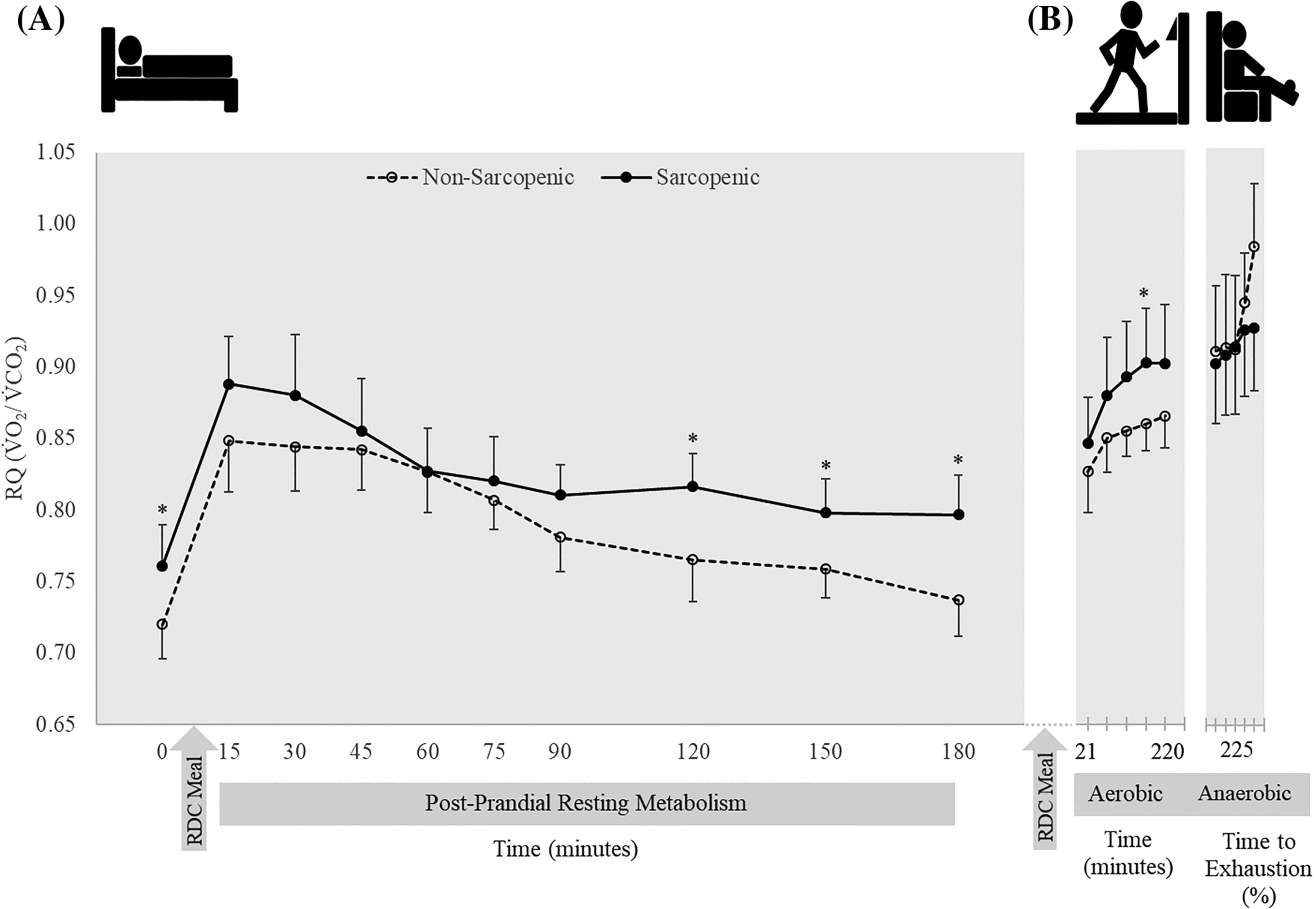
Respiratory quotient (RQ) of 11 nonsarcopenic (NS) (open circles, dashed lines) and 11 sarcopenic (S) (solid circles, solid lines) individuals (A) fasted, postprandial, and during (B) submaximal aerobic exercise and leg extensions to exhaustion. RQ was determined prior to and after consuming a CHO‐rich meal at 15‐ and 30‐min intervals, over 2‐min intervals during aerobic exercise, and over quintiles during anaerobic exercise. Differences between NS and S were determined with independent samples *t*‐tests. * indicates differences between groups (*P* ≤ 0.05). RQ was significantly higher at baseline in S and remained higher at min 120–180. During the submaximal aerobic test, S had a higher RQ than NS from min 6–8. RQ in NS continued to rise during the anaerobic fatiguing task while S had little change from steady‐state aerobic exercise to anaerobic exercise. Values are means ± 95% confidence intervals.

**Figure 4:**
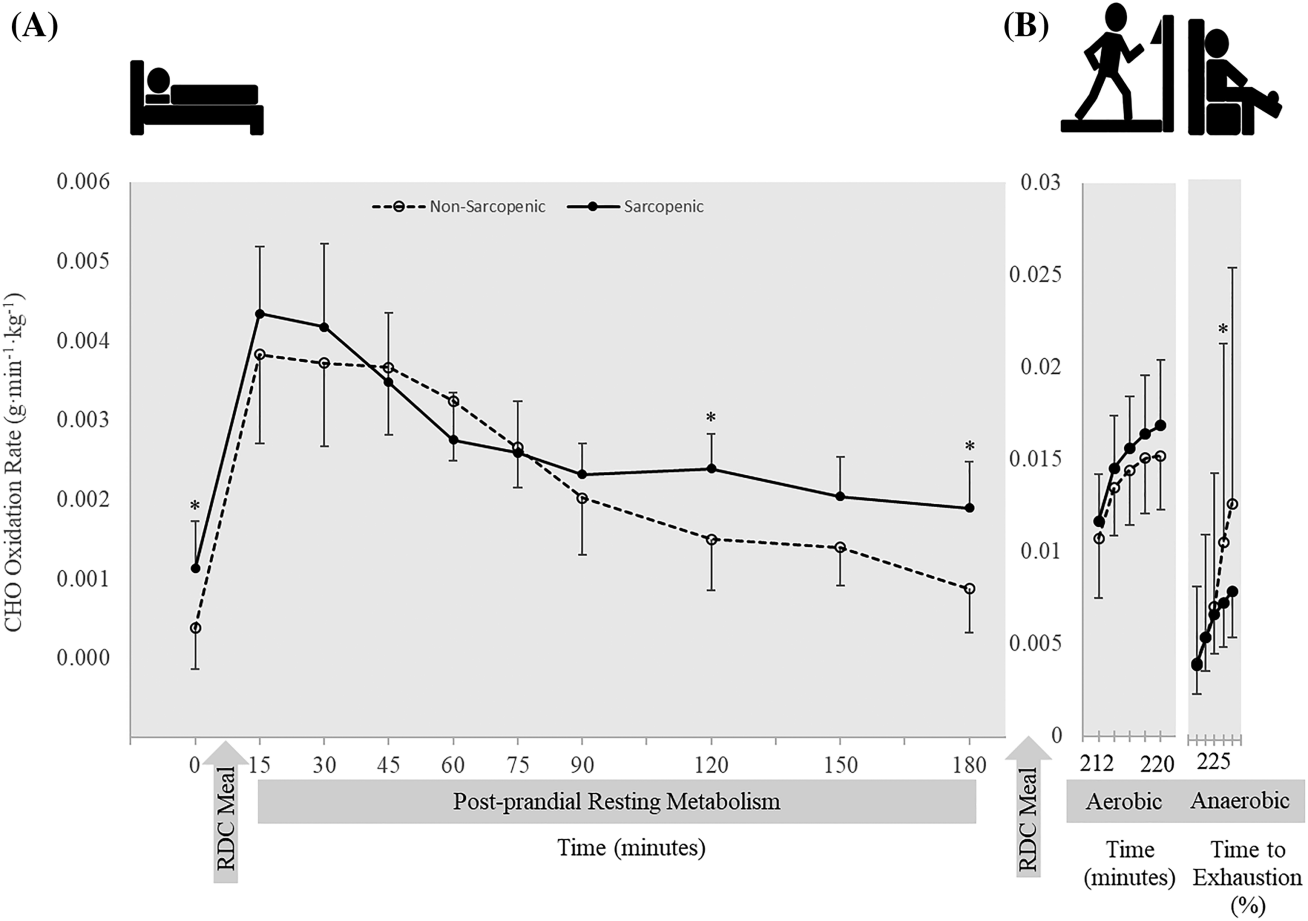
Carbohydrate (CHO) oxidation rate normalized to fat‐free mass of 11 nonsarcopenic (NS) (open circles, dashed lines) and 11 sarcopenic (S) (solid circles, solid lines) individuals (A) fasted, postprandial, and during (B) submaximal aerobic exercise and leg extensions to exhaustion. CHO oxidation was calculated for 30 min prior to and 180 min post‐consumption of a CHO‐rich meal at 15‐ and 30‐min intervals, over 2‐min intervals during aerobic exercise, and over quintiles during anaerobic exercise. Differences between NS and S were determined with independent samples *t*‐tests. * indicates differences between groups (*P* ≤ 0.05). CHO oxidation was greater in S at baseline and at min 120–180. There were no differences between the groups during aerobic exercise. During anaerobic exercise, CHO oxidation was greater in NS at 60–80% time to exhaustion compared with S. Values are means ± 95% confidence intervals.

**Figure 5:**
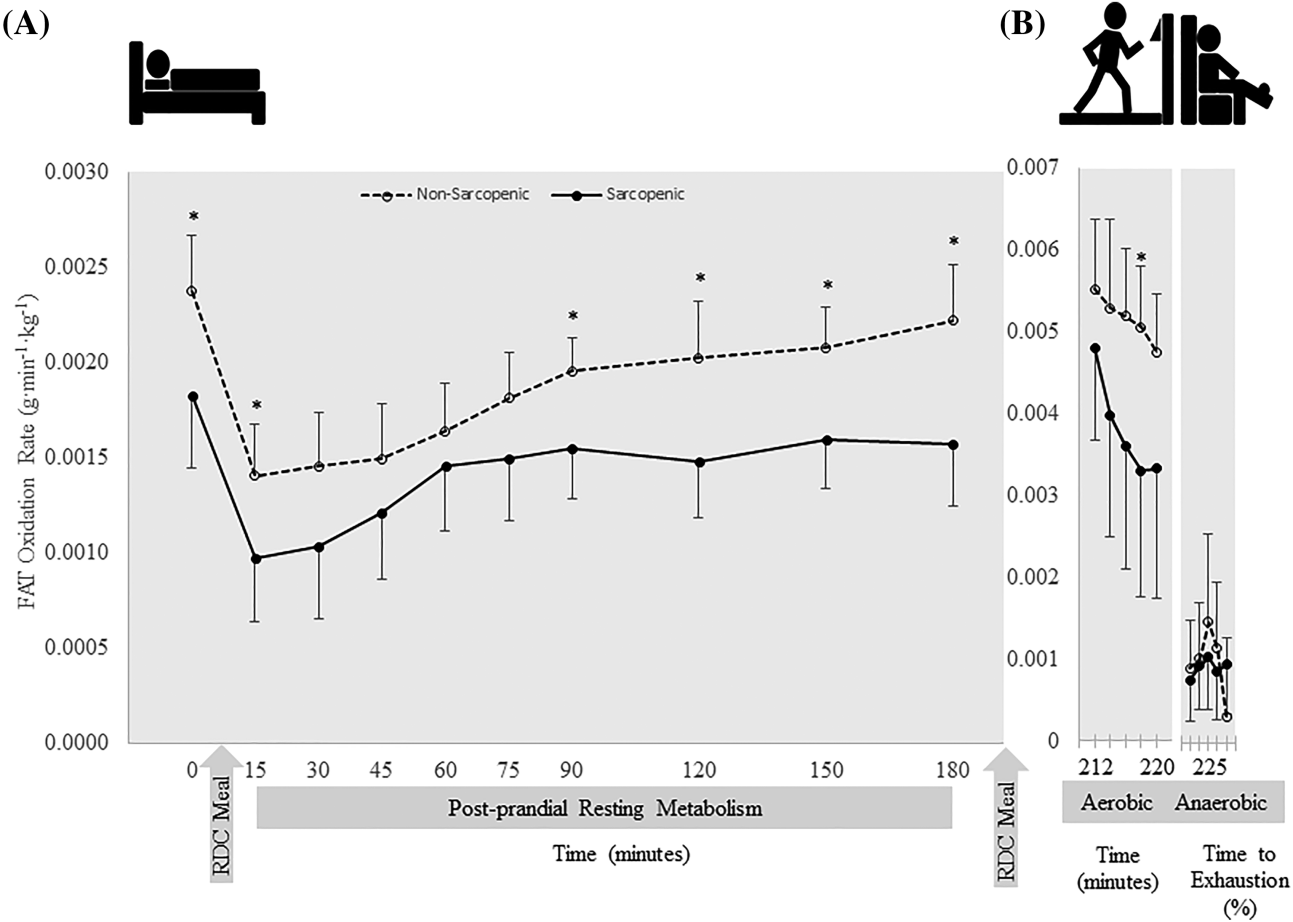
FAT oxidation rate normalized to fat‐free mass of 11 nonsarcopenic (NS) (open circles, dashed lines) and 11 sarcopenic (S) (solid circles, solid lines) individuals (A) fasted, postprandial, and during (B) submaximal aerobic exercise and leg extensions to exhaustion. FAT oxidation was calculated for 30 min prior to and 180 min post‐consumption of a CHO‐rich meal at 15‐ and 30‐min intervals, over 2‐min intervals during aerobic exercise, and over quintiles during anaerobic exercise. Differences between NS and S were determined with independent samples *t*‐tests. * indicates differences between groups (*P* ≤ 0.05). At baseline and throughout the resting period, FAT oxidation was greater in NS compared with S. There were no differences between groups during exercise. Values are means ± 95% confidence intervals.

**Figure 6:**
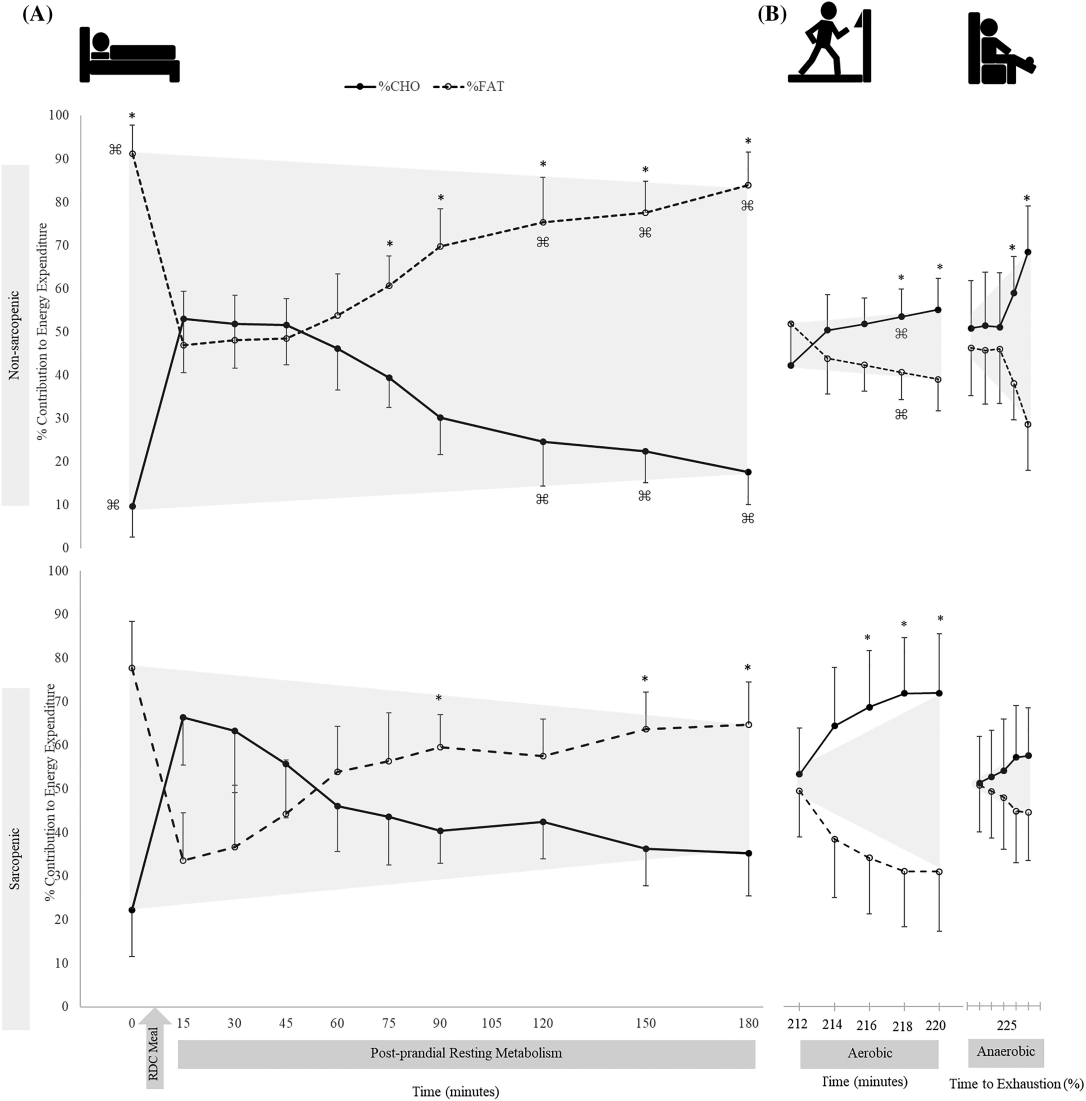
The percent contribution of carbohydrate (CHO) (solid lines) and FAT (dashed lines) utilization to total energy expenditure was calculated during (A) resting and (B) exercise for nonsarcopenic (NS) (top) and sarcopenic (S) individuals (bottom). The percentage of CHO and FAT contribution relative to total energy expenditure was compared between groups using independent samples *t*‐tests. NS showed greater contribution of fat to total energy expenditure at baseline and at 75–180 min and greater CHO oxidation at the min 6–10 of aerobic exercise and 60–100% time to exhaustion of the fatiguing bout. Less variance between CHO and FAT was seen at rest in S, while CHO oxidation was greater at min 4–10 during aerobic exercise. There were no differences between CHO and FAT contributions to substrate utilization in S. * indicates a significant difference between per cent fat and per cent CHO contributions to energy expenditure (*P* ≤ 0.05), and ⌘ indicates a significant difference between S and NS (*P* ≤ 0.05). Values are means ± 95% confidence intervals.

There were no significant time × sex × SS interactions for resting RQ, CHO and FAT oxidation rate, and per cent contribution to energy expenditure for CHO and fat (*P* = 0.739–0.995). There was a time × sex interaction for CHO oxidation (*P* = 0.041) in which female individuals had higher CHO oxidation than male individuals at 15 min (*P* = 0.007), and CHO oxidation was greater at min 45 and 60 than at baseline (*P* = 0.011–0.012). There were no other two‐way interactions (Table S8). There was a significant main effect for SS for RQ, FAT oxidation and per cent contribution of fat (*P* = 0.020–0.024) in which NS individuals demonstrated greater FAT oxidation and per cent contributions from FAT, while S individuals had a higher RQ. Significant main effects for time were present for RQ, CHO and FAT oxidation, and per cent contributions to energy expenditure for CHO and fat (*P* < 0.001) (Table S8). Sarcopenic (S) individuals had a 5–8% higher RQ at baseline (0) (0.76 vs. 072) and at 120, 150 and 180 min (*P* = 0.002–0.025) (Figure 3A) and 59–195% higher CHO oxidation at 0, 120 and 180 min compared with NS (*P* = 0.010–0.047) (Figure 4A). Sarcopenic (S) individuals had 20–31% lower FAT oxidation than NS at 0, 15, 90, 120, 150 and 180 min (*P* = 0.004–0.038) (Figure 5A). Per cent contribution to energy expenditure was greater in S for CHO but lower for FAT at 0, 120, 150 and 180 min (*P* = 0.003–0.046) (Figure 6A).

### Venous blood samples

**Figure 7:**
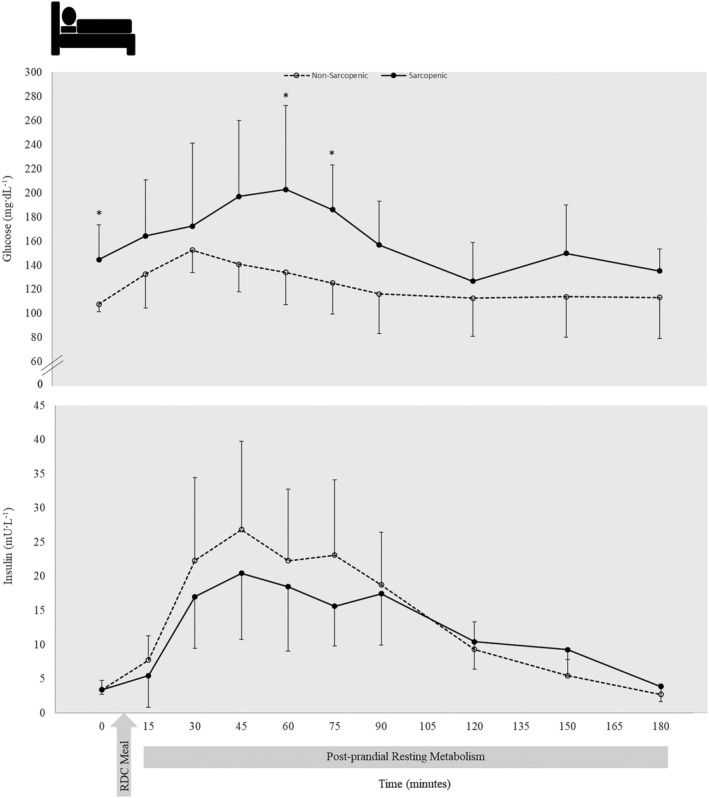
Fasting and postprandial glucose (top) and insulin (bottom) concentrations were measured before and after consumption of a CHO‐rich meal in 10 nonsarcopenic (NS) (open circles, dashed lines) and 9 sarcopenic (S) (solid circles, solid lines) individuals. Blood glucose was significantly higher in S at baseline and remained higher postprandial at 60–75 min. There were no significant differences in insulin levels between NS and S groups. Differences between NS and S were determined with independent samples *t*‐tests. * indicates differences between groups (*P* ≤ 0.05). Values are means ± 95% confidence intervals. Venous blood samples from all timepoints were unavailable for one NS participant, and one NS participant was unable to complete the blood draws at min 30 and 45. Two S participants were able to complete blood draws at 0 and 15 min but were unable to complete the rest of the blood analysis.

There were no significant time × sex × SS interactions for glucose or insulin concentrations. There were significant main effects for time for glucose and insulin, but no main effects for sex or SS (Table S9). Compared with NS, S had higher blood glucose levels at 0, 60 and 75 min, but there were no differences in insulin levels at any timepoint between groups (Figure 7).

### Submaximal aerobic exercise

There were no significant time × sex × SS interactions for RQ, CHO oxidation, FAT oxidation and per cent contribution to energy expenditure of CHO and FAT (*P* = 0.069–0.868). There were no significant time × sex or time × SS interactions (*P* = 0.069–0.914) or main effects for sex or SS (0.094–0.524), but there were significant main effects for time for RQ, CHO oxidation, FAT oxidation and per cent contributions to energy expenditure (*P* < 0.001–0.045) (Table S10). During submaximal aerobic exercise, compared with NS, S individuals had 5% higher RQ at 6–8 min (*P* = 0.039) (Figure 3B), while FAT oxidation was 35% lower at 6–8 min (*P* = 0.033) (Figure 5B). Sarcopenic (S) individuals also displayed a greater per cent contribution to energy expenditure from CHO and a lower contribution from FAT at 6–8 min (*P* = 0.046) (Figure 6B).

### Anaerobic fatiguing exercise

There were no time × sex × SS interactions for RQ, CHO oxidation, FAT oxidation and per cent contributions to energy expenditure of CHO and FAT (*P* = 0.315–0.834). There were no time × sex or sex × SS interactions (*P* = 0.060–0.845). Significant main effects for time were present for RQ, CHO oxidation and per cent contribution of CHO and FAT (*P* < 0.001–0.007) (Table S11). During anaerobic fatiguing exercise, CHO oxidation was 31% greater for NS compared with S at 60–80% (*P* = 0.015) (Figure 4B).

### Correlations

For the entire sample (*n* = 22), RSMI was correlated with RQ at 15 min (*r* = −0.659), CHO oxidation at 15, 30 and 90 min (*r* = −0.719, −0.474 and −0.430, respectively), and FAT oxidation at 15 min (*r* = 0.491). Handgrip strength was correlated with RQ at 15 min (−0.549), and CHO oxidation at 15 and 90 min (*r* = −0.598, −0.552, respectively).

In male individuals (*n* = 11), RSMI was correlated with RQ at 15, 30, 45, 90 and 180 min (*r* = −0.758, −0.634, −0.618, −0.699 and −0.666, respectively); CHO oxidation at 15, 90 and 180 min (*r* = −0.688, −0.715 and −0.633, respectively); and FAT oxidation at 15, 30 and 45 min (*r* = 0.755, 0.716 and 0.616, respectively). Handgrip strength was correlated with RQ at 90 and 180 min (*r* = −0.673, −0.614, −0.754 and −0.668, respectively). In female individuals (*n* = 11), RSMI was correlated with RQ at 120 min (*r* = −0.649) and FAT oxidation at 120, 150 and 180 min (*r* = 0.614, 0.696, 0.611, respectively). Handgrip strength was correlated with RQ at 150 min (*r* = −0.645) and FAT oxidation at 150 min (*r* = 0.637).

## Discussion

There is a plethora of research examining age‐related changes to skeletal muscle including the development of fat infiltration within skeletal muscle, diminished neuromuscular function and muscle atrophy.^6^, ^12^, ^25^, ^26^ While substrate utilization and adaptability to stressors have been studied during exercise in young endurance athletes^27^ and within obese middle‐aged to older adults,^5^, ^28^ less is known in sarcopenia when changing from a fasted to fed state and from rest to exercise. Sarcopenic (S) individuals are known to have lower skeletal muscle mass and strength and also display myosteatosis, which could lead to impairments in metabolic flexibility. However, metabolic flexibility in S individuals has not been investigated before and compared with NS individuals.

To our knowledge, this is the first study to examine differences in metabolic flexibility between NS and S older adults. The main findings of this study indicated that there are distinct differences in skeletal muscle energy metabolism and substrate utilization in S and NS individuals. Differences were observed at rest, after transitioning from a fasted to a fed state, and during exercise. In a fasted state, RQ was higher in S and remained higher postprandially and during exercise, showing less exchange between fuel sources, despite the transition from resting to exercise metabolic stressors (Figure 3). Compared with NS, S individuals demonstrated greater dependency on CHO for energy production during the fasted state. They also had higher CHO oxidation during the late postprandial period after consumption of a CHO‐rich meal, compared with NS individuals who transitioned to an increase in FAT utilization late in the postprandial period (Figures 4, 5, 6). The ability to utilize FAT for fuel was consistently lower in S individuals during the fasted and fed resting state. Even during steady‐state aerobic exercise, although both S and NS individuals utilized CHOs to the same extent, FAT oxidation was significantly lower in S (Figure 5). This points to a dysfunction in the ability of S individuals to process FAT as a fuel, perhaps tied to mitochondrial dysfunction. This could indicate problems with the transport of FAT into the mitochondria or changes in enzymatic pathway efficiencies in conversion of fatty acids into ATP.^29^, ^30^ Because fatty acid oxidation within the mitochondria is typically the primary energy source for muscle during rest or endurance, an impairment of this process could result in muscle fatigue or decreased function.^31^, ^32^ Hyperglycaemia plays a role in reducing mitochondrial fatty acid oxidation, thus blocking FAT transport.^33^ The reduced FAT oxidation, along with higher blood glucose levels after consuming a CHO‐rich meal suggests reduced mitochondrial fat oxidation, indicating impaired metabolic flexibility.^6^, ^34^

Even during the relatively short period (<5 min) of anaerobic exercise‐task to failure, S showed a diminished ability to utilize fuel sources for energy compared with NS, even though they were provided with similar amounts of the meal. Finally, when examining per cent substrate utilization relative to total caloric expenditure, appreciable differences in per cent contributions of CHO and FAT were apparent between NS and S individuals (Figure 6). In culmination, the observed differences in fuel utilization adaptability supports the hypothesis that S individuals are not as metabolically flexible compared with their NS counterparts, which could lead to impairments in generating ATP for muscle function leading to loss of strength over time. In the present study, there were no differences in CHO oxidation between groups during steady‐state aerobic exercise. However, the utilization of CHO increased in NS individuals during fatiguing anaerobic exercise. This suggests that NS were able to respond with an increase in CHO oxidation to generate energy upon demand to match exercise intensity. As exercise intensity increases, skeletal muscle must generate enough energy to meet the demand by switching between fuel sources to generate enough ATP, that is, the muscle must demonstrate metabolic flexibility during exercise to increase support from all available fuel sources to support the exercising muscle.^6^ This process is apparent in the NS individuals as CHO utilization continued to increase during fatiguing exercise. In contrast, the S individuals were unable to further adapt their substrate utilization when challenged with a fatiguing task. In fact, CHO oxidation in the S individuals plateaued and remained similar to levels expressed during submaximal exercise. This could be related to the well‐established age‐related decline in glucose‐fuelled type II muscle fibers^25^ with sarcopenia.

Typically, during a fasted state, fatty acid metabolism predominates as the main energy source.^6^ However, when comparing fasted FAT oxidation between S and NS individuals, S individuals utilized less FAT. It has been suggested that defects in FAT oxidation are associated with insulin resistance and type 2 diabetes^34^ and therefore may be contributory to metabolic inflexibility and disease progression. Even after a high‐CHO meal, NS continued to utilize FAT to a higher degree than seen with sarcopenia. It has been suggested that those with metabolic flexibility may be able to suppress CHO oxidation with an increase in fatty acid oxidation at rest and during exercise, as per the Randle cycle,^35^ which aligns with the greater FAT utilization observed in NS individuals. Even during steady‐state, submaximal exercise, FAT oxidation was consistently higher in the NS individuals. However, during fatiguing high‐intensity exercise, there were no differences in FAT oxidation between groups but greater CHO oxidation in the NS group indicating that NS individuals continued to adapt by switching fuel sources to accommodate the exercise intensity,^27^ while S individuals could not. While there are limitations to measuring substrate utilization during non‐steady‐state exercise, this information still presents trends that are hypothesis‐provoking and warrant further exploration.

The responses for S and NS individuals observed postprandially and during exercise correspond with reported responses in diabetics compared with nondiabetic individuals, respectively,^6^ indicating the possible role of insulin sensitivity with maintaining metabolic flexibility.^34^ We found that S individuals had higher fasted blood glucose levels compared with NS individuals. During the postprandial period, the NS older adults displayed a normal glucose response similar to that reported in healthy younger adults (~39 years)^36^ with a peak in glucose levels occurring 30 min postprandial. On the other hand, S displayed overall higher circulating levels of glucose with a peak in glucose levels occurring later (~60 min postprandial), similar to what has been reported in individuals with type 2 diabetes.^36^ Because skeletal muscle is essential for glucose uptake,^2^ it is possible that the reduced amount of muscle mass due to sarcopenia, as well as muscle insulin resistance, contributed to the delay in glucose uptake after a CHO‐rich meal. Although both groups showed no differences in postprandial circulating insulin levels and HOMA‐IR, S individuals showed a numerical reduction whole‐body insulin sensitivity in comparison with NS individuals, suggesting S individuals are showing early signs of metabolic impairment that could lead to insulin resistance.^31^, ^32^, ^37^ With the differences in insulin sensitivity between S and NS, this suggests the metabolically inflexible NS older adults may be transitioning to an insulin‐resistant state. A more standard glucose clamp test would need to be performed to determine the insulin resistance status of these S individuals.

Additionally, the correlations performed suggest inverse relationships between either RSMI or handgrip strength and RQ and CHO oxidation, and positive relationships between RSMI and FAT oxidation. The greater the muscle mass and strength, the lower the resting RQ and CHO oxidation. Likewise, the greater the muscle mass, the higher the FAT oxidation. Greater muscle mass and strength were generally associated with greater metabolic flexibility and metabolic health. Although these correlations cannot infer causal relationships for all older adults, these correlations help confirm the hypothesis that sarcopenia is associated with metabolic inflexibility.

## Conclusions

Skeletal muscle is critical for movement, but it also plays an essential role in metabolism. As muscle mass, strength and function decline with sarcopenia, the muscle's ability to respond efficiently and effectively to external stressors becomes impaired. The present study demonstrated that S individuals have a diminished ability to adjust muscle fuel utilization to metabolic demand reflecting metabolic inflexibility. These S individuals demonstrated a greater reliance on CHO utilization at rest, indicating an impairment in their ability to utilize FAT as a fuel source. Compared with NS, S had a diminished response in fuel adjustment after consumption of a CHO‐rich meal. Furthermore, metabolic inflexibility became apparent in response to exercise, evidenced by the lack of change in RQ in the S individuals with increasing exercise intensity. Together, these findings indicate that S older adults have an impaired ability to shift fuel utilization in response to different physiological conditions, thus decreasing their energy efficacy, which could lead to long‐term loss of muscle strength and function. Future studies should examine how metabolic flexibility can be improved in the sarcopenic population towards improving strength and function in this population in the long term.

## Conflict of interest

SLP, JMLP and RR are employees and stock holders of Abbott. VAM is a former employee of Abbott. Since 2004, the laboratory directed by JTC has received research grants from Abbott Nutrition, National Cattlemen's Beef Association, Nebraska Beef Council, Nebraska Extension, Stepan Lipid Nutrition, Rock Creek Pharmaceuticals, General Nutrition Corporation, Experimental & Applied Sciences, Nutricia, and/or the USDA National Institute for Food and Agriculture. Between 2008 and 2018, JTC served intermittently as a paid consultant for Regeneron Pharmaceuticals, Abbott Nutrition, General Nutrition Corporation, ErgoGenix/ErgoPharm, or Corr‐Jensen Labs.

## Funding

This work was sponsored by Abbott Nutrition, who contributed to study design, reviewed this manuscript, and made minor edits to the final draft. Abbott Nutrition had no part in data collection, data analysis or interpretations of the findings.

## Supporting information


**Data S1.** Inclusion and exclusion criteria met by participants in order to be eligible for the Test Visit.

**Data S2.** Strength and function assessments performed by participants in the Screening Visit to determine eligibility and classification of sarcopenic status.

**Data S3.** Methodology of the anthropometric and body composition assessments to determine eligibility and classification of sarcopenic status in non‐sarcopenic (NS) (*n* = 11) and sarcopenic (S) (n = 11) older adults.

**Data S4.** Methodology the assessment of leg extension strength in nonsarcopenic (NS) (n = 11) and sarcopenic (S) (*n* = 11) older adults.

**Data S6.** Methodology of indirect calorimetry and metabolic measurements performed at rest and during exercise in non‐sarcopenic (NS) (*n* = 11) and sarcopenic (S) (*n* = 11) older adults.

**Data S7.** Methodology of the venous blood samples collected during the Test Visit in non‐sarcopenic (NS) (n = 11) and sarcopenic (S) (n = 11) older adults.

**Table S1.** Inclusion and exclusion criteria met by participants in order to be eligible for the Test Visit.

**Table S2.** Strength and function assessments performed by participants in the Screening Visit to determine eligibility and classification of sarcopenic status.

**Table S5.** Means ± standard deviations (SD) of energy intake and macronutrient composition of food consumed the three days prior to the experimental visit. P‐values are type I errors of the interactions and main effects.

**Table S8.** Respiratory Quotient (RQ) and substrate utilization at baseline and 180 minutes post‐prandial a CHO‐rich meal. Values are means ± standard deviations (SD). P‐values are type I errors of independent t‐tests.

**Table S9.** Venous blood samples taken at baseline and 180 minutes postprandial a CHO‐rich meal. Values are means ± standard deviations (SD). P‐values are type I errors of independent t‐tests.

**Table S10.** Substrate utilization during a submaximal aerobic test at 50–60% of estimated V̇O2. Values are means ± standard deviations (SD). P‐values are type I errors of independent t‐tests.

**Table S11.** Substrate utilization during an anaerobic fatiguing test at 30% of estimated one‐repetition maximum. P‐values are type I errors of independent t‐tests.
