# Supplementary material for: Differences in muscle energy metabolism and metabolic flexibility between sarcopenic and nonsarcopenic older adults
Source: J Cachexia Sarcopenia Muscle. 2022 Feb 17;13(2):1224–37. doi: 10.1002/jcsm.12932 (PMC8978004; doi:10.1002/jcsm.12932)
Supplement: Supplementary file 1 — Data S1. Inclusion and exclusion criteria met by participants in order to be eligible for the Test Visit. [file JCSM-13-1224-s001.pdf]

Appendix 1. Study Protocol NCT03701867

ABBOTT NUTRITION, ABBOTT LABORATORIES

RESEARCH & DEVELOPMENT, SCIENTIFIC AND MEDICAL AFFAIRS

3300 Stelzer Rd, Columbus, OH 43219

STUDY PROTOCOL

Study Title: A pilot study to explore muscle energy metabolism and metabolic flexibility in older men and women.

Study Site: University of Nebraska

Investigators: Joel Cramer, Ph.D.

ClinicalTrials.gov Identifier: NCT03701867

Actual Enrollment: 22 participants

Intervention Model: Single Group Assignment

Masking: None (Open Label)

Actual Study Start Date: November 14, 2018

Actual Primary Completion Date: July 22, 2019

Actual Study Completion Date: July 22, 2019

STUDY OBJECTIVES

To explore muscle energy metabolism and metabolic flexibility under various conditions in older men and women having normal and low muscle mass/strength/performance. This is a pilot study to explore differences in metabolic flexibility (e.g., carbohydrate and fat metabolism) at rest, after a meal, and during exercise in sarcopenic vs. nonsarcopenic older men and women.

OVERVIEW OF STUDY PROTOCOL:

Ages Eligible for Study: 65 Years and older (Older Adult)

Sexes Eligible for Study: All

Accepts Healthy Volunteers: Yes

Inclusion Criteria:

- Body mass index (BMI) >20.0 and <39.0 kg/m2
- Ambulatory (may use assistance device e.g., cane, walker)
- Not a current smoker (within past 10 years)
- Low or moderate risk based on the responses from the AHA/ACSM Health/Fitness Facility Preparticipation Screening Questionnaire
- Normal muscle mass and strength/performance or sarcopenia included in low muscle mass and low grip strength
- If on thyroid medication or hormone replacement therapy, has been on a constant dosage for at least 2 months prior to Screening Visit
- Willingness to follow protocol as described
- Voluntarily signed and dated an informed consent form (ICF), approved by an Independent Ethics Committee (IEC)/Institutional Review Board (IRB) prior to any participation in the study

Exclusion Criteria:

- Active/treated disease, under the care of a physician, for the following: metabolic/endocrine (diabetes), hepatic, or renal disease, myocardial infarction, peripheral vascular disease, respiratory or neuromuscular disease
- Participates in a resistance exercise program
- Poor appetite with recent unexplained weight loss over the past 6 months
- Current infection (requiring medication or which might be expected to require hospitalization); has had inpatient surgery, or corticosteroid treatment (excluding topical creams) in the last 3 months or antibiotics in the last 3 weeks.
- Active malignancy, excluding carcinoma in-situ of the cervix, cutaneous malignancies (basal cell carcinoma, squamous cell carcinoma, except melanoma)
- Chronic, contagious, infectious disease, such as active tuberculosis, Hepatitis A, B or C, or HIV
- Taking medications/dietary supplements or substances that could profoundly modulate metabolism in the opinion of the principal investigator or study physician, Exceptions for multi-vitamin/mineral supplement, topical or optical steroids and short-term use (less than two weeks) of dexamethasone

- Allergy or intolerance to any foods
- History of gastrointestinal disease, or surgeries, gastroparesis, or taking medications that are known in the opinion of the PI or study physician to interfere with consumption/digestion/absorption of nutrients
- Eating disorder, severe dementia or delirium, history of significant neurological or psychiatric disorder, alcoholism, substance abuse or other conditions that may interfere with compliance with study protocol procedures in the opinion of the principal investigator or study physician
- Participant in a concomitant AN trial or trial of a non-registered drug (or is within the 30 day follow-up period for such a trial) or that otherwise conflicts with this study unless otherwise approved.

After a screening visit, eligible subjects will be scheduled for a single study test visit to assess carbohydrate and fat oxidation at rest, during aerobic and fatiguing muscle strength/endurance tests, and after a standard meal. Additionally, body composition via (dual x-ray absorptiometry, Screening Visit)) and venous blood samples will be obtained for assessment of metabolic fuels (glucose, free fatty acids), amino acids, and hormones that are known to be important for their uptake in muscle.

Standard Meal:

Gatorade™ Classic. Beverages are flavored, noncarbonated, caffeine-free and available in 34 g carbohydrate from sugar and dextrose.

Additional Foods to be provided to subject after initial volume (10 oz) of Gatorade consumed first.

Morning- ½ English Muffin, regular:	70 calories, 1 g fat, 12 g CHO, 2 g protein
1 Tbl Peanut Butter:	94 calories, 8 g fat, 3 g CHO, 4 g protein
Gatorade:	140 calories, 0 g fat, 36 g CHO, 0 g protein
TOTAL:	304 calories, 9 g fat, 51 g CHO, 6 g protein

Lunch- 1 slice white bread:	80 calories; 4 g fat; 12 g CHO; 2 g protein
1 oz American cheese:	96 calories; 5 g fat; 2 g CHO; 4 g protein
Gatorade:	140 calories, 0 g fat, 36 g CHO, 0 g protein
TOTAL:	316 calories, 9 g fat, 50 g CHO, 6 g protein

Overview of study schedule:

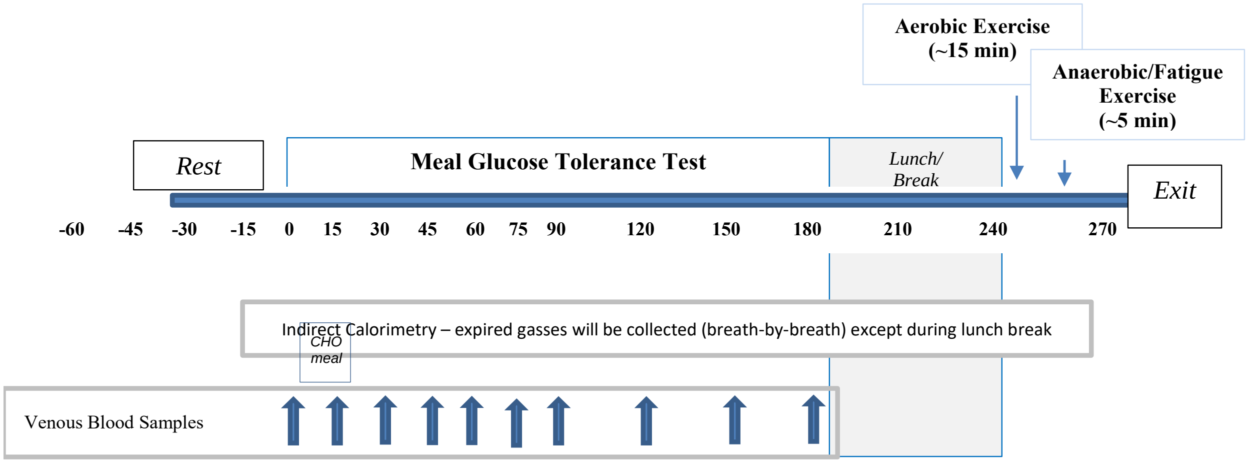

Outcome Measures

Primary Outcome Measures:

Respiratory Quotient [Time Frame: Baseline to 270 minutes]

Ratio of macronutrient oxidation

Secondary Outcome Measures:

Blood Biochemistries - Glucose [Time Frame: Baseline to 270 minutes]

Venous blood sample

Blood Biochemistries - Insulin [Time Frame: Baseline to 270 minutes]

Venous blood sample

Blood Biochemistries - Amino Acids [Time Frame: Baseline to 270 minutes]

Venous blood sample

Blood Biochemistries - Fatty Acids [Time Frame: Baseline to 270 minutes]

Venous blood sample

Muscle Oxygenation [Time Frame: Baseline to 270 minutes]

Near Infrared Spectroscopy

Muscle Fatigue [Time Frame: Baseline to 270 minutes]
Electromyography

	Screening Visit[1]	***Study Test Visit***[2]
Informed Consent/HIPAA	**X**	
Age, Height, Weight, Waist Circumference	**X**	
Pre-exercise risk stratification via AHA/ACSM Health/Fitness Facility Preparticipation Screening Questionnaire[3]	**X**	
Medical History	**X**	
Medications (and dietary supplements)	**X**	
Grip Strength[4]	**X**	
Short Physical Performance Battery (including normal gait speed)[5]	**X**	
Body Composition[6]	**X**	
Non-exercise Estimation of VO_2max; 5RM estimation of 1RM leg (knee) extension strength[7]	**X**	
Fasting blood sample[8]		**X**

Oral meal glucose tolerance test with blood sampling,[9] and indirect calorimetry [10]		X
Standard Lunch[11]		X
Exercise tests with indirect calorimetry[12]		X
Adverse Events[13]	X	X

Schedule of Study Visits:

1. Screening assessments to determine eligibility may be conducted on separate days.
2. Study test visit to be scheduled within approximately 10 days of screening visit.
3. Four-step risk stratification via AHA/ACSM Health/Fitness Facility Preparticipation Screening Questionnaire and Physical Activity Readiness Questionnaire
4. Grip Strength (Jamar handgrip dynamometer)
5. Short Physical Performance Battery
6. Classifying Hi & Low Muscle Mass will be assessed by dual x-ray absorptiometry, but only in potentially-eligible subjects.
7. Non-exercise Estimation of VO_2max and submaximal 5RM estimation of 1RM leg (knee) extension strength.
8. Fasting blood sample to be analyzed for glucose, hormones.
9. Oral meal glucose tolerance test includes venous blood sample at Time 0 followed by consumption of a standard meal containing approximately 50 g carbohydrate within 15 minutes followed by venous blood samples approximately every 15 $\pm$ 5 min up to 90 minutes, then approximately every 30 $\pm$ 5 minutes up to 180 minutes, to be analyzed for glucose, free fatty acids, amino acids, insulin, c-peptide, GLP-1, GIP.
10. Indirect calorimetry to measure the difference between inspired and expired volumes of O_2 and CO_2 will be collected breath-by-breath throughout the resting and exercise states for analysis of O_2 and CO_2 (ParvoMedics TrueOne 2400 Metabolic Cart, http://www.parvo.com/trueone-2400/).
11. Standard lunch to include approximately 50 g carbohydrate. Subject will consume standard lunch within approximately 30 minutes followed by approximately 30-minute rest.
12. Submaximal exercise tests to assess low-moderate intensity aerobic metabolism and submaximal anaerobic strength-endurance will be performed by a) walking on a treadmill at 50% to 60% of the estimated VO_2max for a total of 10 minutes, followed by b) unilateral leg (knee) extension exercises at 50% of estimated 1RM to volitional exhaustion, respectively.
13. Non-serious and serious AEs will be collected from the time the informed consent form is signed until study exit.

STATISTICAL METHODS

The primary aim is to investigate potential differences between sarcopenic and non-sarcopenic groups via a set of Confidence Intervals, with the 95% as well as 80% and 70% Confidence Intervals to be examined.

The primary analysis will include all available subject's data.

Sample Size

There will be approximately 10 men and women (50:50) enrolled in each group. Based on similar studies in the literature (Roberts, SB., and Rosenberg I. Physiol Rev 86: 651–667, 2006; doi:10.1152/physrev.00019.2005), this proposed sample size should provide a directional understanding of the differences between groups.
