# Supplementary material for: Differences in muscle energy metabolism and metabolic flexibility between sarcopenic and nonsarcopenic older adults
Source: J Cachexia Sarcopenia Muscle. 2022 Feb 17;13(2):1224–37. doi: 10.1002/jcsm.12932 (PMC8978004; doi:10.1002/jcsm.12932)
Supplement: Supplementary file 2 — Data S2. Strength and function assessments performed by participants in the Screening Visit to determine eligibility and classification of sarcopenic status. [file JCSM-13-1224-s002.pdf]

Appendix 2: Statistical Analysis Plan

STATISTICAL METHODS

Protocol Number:	BL39
Protocol Title:	A pilot study to explore muscle energy metabolism and metabolic flexibility in older men and women
Study Sponsor:	Abbott Nutrition
Study Site:	University of Nebraska
Investigators:	Joel Cramer, Ph.D.

Statistical and Analytical Plans

This is a cross-sectional pilot study to explore differences in metabolic flexibility (e.g., carbohydrate and fat metabolism) at rest, after a meal, and during exercise in sarcopenic vs. nonsarcopenic older men and women. Approximately 24 subjects will be enrolled (50:50, male: female and sarcopenic: nonsarcopenic), to finish a minimum of 20 subjects.

Each continuous variable in the crossover design will be evaluated using parametric or nonparametric (if parametric assumptions do not hold) analysis. The residuals from the parametric analysis will be utilized to check for validity of the parametric test assumptions.

Parametric:

The parametric analysis will be performed using two-way (sex, sarcopenia) between-subjects ANOVAS and mixed-factorial ANOVAS with between-subjects factors (sex, sarcopenia) and within-subjects factor (time) with appropriate covariance structure (e.g., compound symmetry covariance structure) and appropriate degrees of freedom (e.g., Kenward-Roger, Sattherwaite, etc.).

Nonparametric:

If the parametric approach is determined to be inappropriate, then a two-sided Wilcoxon Rank Sum test will be used.

Variable Definition:

Using O_2 and CO_2 data, area under the curve (AUC) and positive AUC will be calculated. An observation having both time and value will be called non-missing point. Otherwise it will be called missing point. A non-missing point within ±5 minutes of the planned time will be called valid point. Otherwise it will be called invalid point. Specifications for various calculations are given below.

Area under the curve (AUC):

Trapezoidal AUC from 0 to 180 minutes for O_2 and CO_2 gases will be calculated using planned times (0 and every 15 minutes for up to 180 minutes) with its valid or linearly interpolated points according to the following rules (baseline value, collected immediately before consumption of the study product, will be used for the zero-minute value):

- AUC from 0 to 180 minutes will be missing if there are less than 7 (allowing 6 invalid points in 0 to 180 minutes) valid points with at least one valid point every 30 minutes.
- AUC from 0 to 180 minutes will be missing if the time-zero point is missing.
- AUC from 0 to 180 minutes will be missing if the end point is missing.

If AUC from 0 to 180 minutes is not declared missing by the above rules, then proceed to next point.

- If a point at the planned time **t**, $15 \leq \mathbf{t} \leq 180$ minutes, is invalid then two nonmissing points nearest to time **t**, before and after **t**, will be used to linearly interpolate at time **t** to calculate the AUC.
- If the end point at 180 minutes is invalid then use two nonmissing points nearest to 180 minutes, before and after 180 minutes, with distance less than or equal to $2 \times [180 - (165 - 5)]$ minutes to linearly interpolate at 180 minutes to calculate the AUC.

Invalid endpoint before 180 – 5 minutes will not be extrapolated.

For blood sample variables, since there are less collection times, all values must be valid points.

Positive AUC:

Positive AUC from 0 to 180 minutes will be calculated by adding up only the portions of the AUC that are above the value at time-zero. The portions of the AUC that are below the value at time-zero will be counted as zero.

The primary aim is to investigate potential differences between sarcopenic and non-sarcopenic groups via a set of confidence intervals, with the 95% as well as the 80% and 70% confidence intervals to be examined.

Statement of Primary Hypothesis (Hypotheses)

There is no primary variable for this pilot; however, how metabolic flexibility may differ between groups is of primary interest and will be assessed under different physiologic conditions: i) at rest, ii) during aerobic exercise and anaerobic fatiguing exercise, and iii) after meal glucose tolerance test; *adjusted and unadjusted for fat-free mass*. The following variables are of interest:

- Fat oxidation (%)
- Carbohydrate Oxidation (%)
- Respiratory Quotient (RQ) or Respiratory Exchange Ratio (RER; VCO_2/CO_2)
- Number of repetitions to failure
- HOMA-IR (fasting insulin mU/L*fasting glucose mg/dL/405)
- Glucose (AUC and peak)
- Insulin (AUC and peak)

Meaningful outcome(s) would be to observe differences between sarcopenic status at rest, after a meal, and during exercise in: 1) fat oxidation, 2) carbohydrate oxidation, 3) RQ, 4) muscle oxygenation, 5) glucose and insulin

Data Sets to be Analyzed

The primary analysis will include all available subject's data. Raw scores and changes from baseline will be analyzed.

Hypothesis Testing

As this is not a hypothesis testing study, statistical tests will be conducted suitable for cross-sectional study design using alpha = 0.20 level tests for indication of a "trend" and will need to be interpreted with caution. The primary aim is to investigate the potential differences via a set of confidence intervals, with the 95%, as well as the 80% and 70% confidence intervals to be examined.

Missing, Spurious, or Unused Data

Summaries will be generated for the purpose of identifying potential spurious observations (values). If apparently extreme observations (values) are confirmed, then sensitivity analysis may be made to determine the effect of the extreme observations (values). If the results of the two analyses differ, this will be discussed in the reporting of statistical results. Any data not used will be described.

Interim Analysis Plan

No interim analyses are planned.

Criteria for Early Termination of the Trial

There are no plans to terminate the trial early.

Serious Adverse Events

All AEs and SAEs that are collected will be summarized with data listings and summary statistics.

Adjustment of Significance and Confidence Levels

There will be no adjustment made to significance and confidence levels in this pilot trial.

Adjustments for Covariates

Chronological age, muscle mass, and fat mass are potential covariates. If additional covariates are used, they will be listed in the report.

Transformations

If data transformations (such as logarithmic, arcsine of the square root) are used to improve distribution characteristics (normality, variance homogeneity) for parametric procedures, they will be described in reporting of statistical results.

Other Considerations

If any changes to the statistical analysis plan are deemed necessary, or post-hoc analysis are done, these will be described in detail in the statistical report.

Exit Reasons

Reasons for exiting the study and adverse events will be summarized with summary statistics.

Determination of Sample Size

There will be approximately 10 men and women (50:50) enrolled in each group. This proposed sample size should provide directional understanding of the differences between groups.
