# Supplementary material for: Differences in muscle energy metabolism and metabolic flexibility between sarcopenic and nonsarcopenic older adults
Source: J Cachexia Sarcopenia Muscle. 2022 Feb 17;13(2):1224–37. doi: 10.1002/jcsm.12932 (PMC8978004; doi:10.1002/jcsm.12932)
Supplement: Supplementary file 3 — Data S3. Methodology of the anthropometric and body composition assessments to determine eligibility and classification of sarcopenic status in non‐sarcopenic (NS) (n = 11) and sarcopenic (S) (n = 11) older adults. [file JCSM-13-1224-s010.pdf]

Differences in Muscle Energy Metabolism and Metabolic Flexibility between Sarcopenic and Non-sarcopenic Older Adults, Journal of Cachexia, Sarcopenia and Muscle.

Marni E. Shoemaker, Suzette L. Pereira, Vikkie A. Mustad, Zachary M. Gillen, Brianna D. McKay, Jose M. Lopez-Pedrosa, Ricardo Rueda, Joel T. Cramer*

* College of Health Sciences, The University of Texas at El Paso, El Paso, TX 79968, USA, jtcramer@utep.edu

Supporting Information S3. Methodology of the anthropometric and body composition assessments to determine eligibility and classification of sarcopenic status in non-sarcopenic (NS) (n=11) and sarcopenic (S) (n=11) older adults.

Anthropometric measurements were taken for height (cm) and body mass (kg) using a calibrated digital scale and stadiometer (Seca 769, Hamburg, Germany). Body mass index (BMI) was calculated, and waist circumference was measured. Fat mass (FM, kg), fat-free mass (FFM, kg), percent body fat (BF%), and appendicular lean soft tissue (ALST) were assessed using whole-body DXA (Lunar iDXA, GE Healthcare, Madison, WI). The DXA was calibrated daily (Lunar iDXA User Manual, GE Healthcare, Madison, WI). Participants were instructed to lie supine on the padded scanner table with their hands pronated and near their body. Participants were categorized as "normal" or "low" skeletal muscle mass based on relative skeletal muscle index percent (RSMI%) from Kim et al.[14] and Janssen et al.[15]
