# Supplementary material for: Differences in muscle energy metabolism and metabolic flexibility between sarcopenic and nonsarcopenic older adults
Source: J Cachexia Sarcopenia Muscle. 2022 Feb 17;13(2):1224–37. doi: 10.1002/jcsm.12932 (PMC8978004; doi:10.1002/jcsm.12932)
Supplement: Supplementary file 4 — Data S4. Methodology the assessment of leg extension strength in nonsarcopenic (NS) (n = 11) and sarcopenic (S) (n = 11) older adults. [file JCSM-13-1224-s005.pdf]

Differences in Muscle Energy Metabolism and Metabolic Flexibility between Sarcopenic and Non-sarcopenic Older Adults, Journal of Cachexia, Sarcopenia and Muscle.

Marni E. Shoemaker, Suzette L. Pereira, Vikkie A. Mustad, Zachary M. Gillen, Brianna D. McKay, Jose M. Lopez-Pedrosa, Ricardo Rueda, Joel T. Cramer*

* College of Health Sciences, The University of Texas at El Paso, El Paso, TX 79968, USA, jtcramer@utep.edu

Supporting Information S4. Methodology the assessment of leg extension strength in non-sarcopenic (NS) (n=11) and sarcopenic (S) (n=11) older adults.

Maximal leg extension strength was estimated sub-maximally with a unilateral, dynamic constant external resistance 5RM test.[18] Leg extensions were completed on a plate-loaded leg extension machine (Hammer Strength Plate-Loaded, Iso-Lateral Leg Extension Machine; LifeFitness, Rosemont, IL, USA) that was custom fitted with a load cell (Omegadyne, model LCHD-500, 0-500 lb; Stamford, CT, USA). Participants were seated on a Biodex chair (Biodex Medical Systems, Inc., Shirley, NY, USA) and secured with restraining straps over the pelvis, trunk, and contralateral thigh. Participants were instructed to sit upright with their back against the chair and to tightly hold the handles located near their hips. The lateral epicondyle of the right femur was aligned with the axis of rotation of the leg extension device.

A brief warm-up set of 10 repetitions with 4.5 kg external resistance was performed first to familiarize the participants with the leg extension movement. After a two-min rest period, an appropriate amount of weight was added for a first attempt to find a 5-RM. Two to five min rest was allowed between attempts. The set was determined as a successful 5-RM if the participant

was able to complete the five repetitions through their full range of motion, but not able to complete a sixth repetition.
