# Supplementary material for: Differences in muscle energy metabolism and metabolic flexibility between sarcopenic and nonsarcopenic older adults
Source: J Cachexia Sarcopenia Muscle. 2022 Feb 17;13(2):1224–37. doi: 10.1002/jcsm.12932 (PMC8978004; doi:10.1002/jcsm.12932)
Supplement: Supplementary file 5 — Data S6. Methodology of indirect calorimetry and metabolic measurements performed at rest and during exercise in non‐sarcopenic (NS) (n = 11) and sarcopenic (S) (n = 11) older adults. [file JCSM-13-1224-s011.pdf]

Differences in Muscle Energy Metabolism and Metabolic Flexibility between Sarcopenic and Non-sarcopenic Older Adults, Journal of Cachexia, Sarcopenia and Muscle.

Marni E. Shoemaker, Suzette L. Pereira, Vikkie A. Mustad, Zachary M. Gillen, Brianna D. McKay, Jose M. Lopez-Pedrosa, Ricardo Rueda, Joel T. Cramer*

* College of Health Sciences, The University of Texas at El Paso, El Paso, TX 79968, USA, jtcramer@utep.edu

Supporting Information S6. Methodology of indirect calorimetry and metabolic measurements performed at rest and during exercise in non-sarcopenic (NS) (n=11) and sarcopenic (S) (n=11) older adults.

The rate of oxygen consumption ($\dot{V}O_2$) and carbon dioxide production ($\dot{V}CO_2$) were measured continuously with calibrated metabolic carts (Parvo Medics TrueOne® 2400 Metabolic Measurement System, Sandy, Utah). The oxygen and carbon dioxide gas analyzers were calibrated with a gas mixture of 4% CO_2, 16% O_2, and 80% N. The spirometer was calibrated with a 3 L syringe (Series 5530, Hans Rudolf, Inc., Shawnee, KS). During resting measurements, a ventilated metabolic hood (Parvo Medics TrueOne® 2400 Canopy System, Sandy, Utah) was placed over the head while the participant was lying supine on a bed. The metabolic hood was secured to form an airtight seal around the head. During the aerobic and anaerobic exercise tests, face masks (7450 V2, Hans Rudolph, Inc., Shawnee, KS, size small - large), headgear (7450 V2, Hans Rudolph, Inc., Shawnee, KS), with attached two-way non-rebreathing valves (2700, Hans Rudolph, Inc., Shawnee, KS) were secured around the nose and mouth of each participant to form an air tight seal on the face. Participants were refrained from talking during all metabolic testing procedures.
