# Supplementary material for: Differences in muscle energy metabolism and metabolic flexibility between sarcopenic and nonsarcopenic older adults
Source: J Cachexia Sarcopenia Muscle. 2022 Feb 17;13(2):1224–37. doi: 10.1002/jcsm.12932 (PMC8978004; doi:10.1002/jcsm.12932)
Supplement: Supplementary file 6 — Data S7. Methodology of the venous blood samples collected during the Test Visit in non‐sarcopenic (NS) (n = 11) and sarcopenic (S) (n = 11) older adults. [file JCSM-13-1224-s013.pdf]

Differences in Muscle Energy Metabolism and Metabolic Flexibility between Sarcopenic and Non-sarcopenic Older Adults, Journal of Cachexia, Sarcopenia and Muscle.

Marni E. Shoemaker, Suzette L. Pereira, Vikkie A. Mustad, Zachary M. Gillen, Brianna D. McKay, Jose M. Lopez-Pedrosa, Ricardo Rueda, Joel T. Cramer*

* College of Health Sciences, The University of Texas at El Paso, El Paso, TX 79968, USA, jtcramer@utep.edu

Supporting Information S7. Methodology of the venous blood samples collected during the Test Visit in non-sarcopenic (NS) (n=11) and sarcopenic (S) (n=11) older adults.

Approximately 12 mL of blood was collected at each timepoint and separated into serum and plasma vacutainer tubes (Fisher Scientific). Samples were centrifuged at 2000 g for 15 min, aliquoted into microtubes, and stored in a -80°C freezer for later analysis. Plasma was collected with Heparin anticoagulant and analyzed in duplicate by enzyme-linked immunosorbent assays (ELISA) to determine concentrations of insulin ($mU \cdot L^{-1}$) (Insulin ELISA kit, Crystal Chem High Performance Assays) per kit instructions. Serum was collected and analyzed for glucose concentrations ($mg \cdot dL^{-1}$) with a Vitros 250 Chemistry Analyzer (Ortho-Clinical Diagnostics).
