# Supplementary material for: Differences in muscle energy metabolism and metabolic flexibility between sarcopenic and nonsarcopenic older adults
Source: J Cachexia Sarcopenia Muscle. 2022 Feb 17;13(2):1224–37. doi: 10.1002/jcsm.12932 (PMC8978004; doi:10.1002/jcsm.12932)
Supplement: Supplementary file 7 — Table S1. Inclusion and exclusion criteria met by participants in order to be eligible for the Test Visit. [file JCSM-13-1224-s004.pdf]

Differences in Muscle Energy Metabolism and Metabolic Flexibility between Sarcopenic and Non-sarcopenic Older Adults, Journal of Cachexia, Sarcopenia and Muscle.

Marni E. Shoemaker, Suzette L. Pereira, Vikkie A. Mustad, Zachary M. Gillen, Brianna D. McKay, Jose M. Lopez-Pedrosa, Ricardo Rueda, Joel T. Cramer[*]

[*] College of Health Sciences, The University of Texas at El Paso, El Paso, TX 79968, USA, jtcramer@utep.edu

Supplementary Table S1. Inclusion and exclusion criteria met by participants in order to be eligible for the Test Visit.

**Inclusion Criteria**	
	Participant is 65 years of age or older at the time of screening
	Participant's body mass index (BMI) is ≥ 18.0 and ≤ 39.0 $kg \cdot m^{-2}$
	Participant is ambulatory (able to walk without assistance)
	Participant is not a current smoker (within past 10 years)
	Participant is classified as low OR moderate risk as defined by the American College of Sports Medicine (ACSM) Guidelines for Exercise Testing & Prescription[1] based on the responses from AHA/ACSM Health/Fitness Facility Preparticipation Screening Questionnaire
	Participant has normal muscle mass and strength/performance (normal grip strength [≥ 30.0 kg (men); ≥ 20.0 kg (women)]) (NS) **OR** low muscle mass and strength/performance (low grip strength [< 30 kg (men); < 20 kg (women)]) (S) according to revised EUGSOP definition of sarcopenia;[2]
	If Participant is on thyroid medication or hormone replacement therapy,

states he/she has been on a constant dosage for at least 2 months prior to Screening Visit

Participant states he/she is willing to follow protocol as described

Participant has voluntarily signed the Informed Consent Form

Exclusion Criteria

Participant states he/she has a history of metabolic/endocrine (diabetes), hepatic, or renal disease, myocardial infarction, peripheral vascular disease, respiratory or neuromuscular disease

Participant states he/she regularly participates in a resistance exercise program

Participant states he/she has had poor appetite with recent unexplained weight loss (e.g., 10 pounds [4.5 kilograms]) over the past 6 months

Participant states he/she has a current infection (requiring medication or which might be expected to require hospitalization), has had inpatient surgery, or corticosteroid treatment (excluding topical creams) in the last 3 months or antibiotics in the last 3 weeks prior to the Screening Visit

Participant states that he/she has an active malignancy, excluding carcinoma in-situ of the cervix, cutaneous malignancies (basal cell carcinoma, squamous cell carcinoma, except melanoma)

Participant states that he/she has a chronic, contagious, infectious disease, such as active tuberculosis, Hepatitis A, B, or C, or HIV

Participant reports currently taking medications/dietary supplements or substances that could profoundly modulate metabolism in the opinion of the

principal investigator (PI) or study physician, e.g. progestational agents, steroids, growth hormone, dronabinol, marijuana, CaHMB, free amino acid supplements, dietary supplements to aid weight loss or gain. Exceptions included use of multi-vitamin/mineral supplement, topical or optical steroids and short-term use (less than two weeks) of dexamethasone

Participant is known to be allergic or intolerant to any foods

Participant states he/she has had history of gastrointestinal disease (e.g., Crohn's, colitis, celiac), or surgeries (including gastric balloon), gastroparesis, or taking medications that are known in the opinion of the PI or study physician (e.g., cholinergic agonists, prokinetic agents, opioid antagonists, antidiarrheals, and antibiotics) to interfere with consumption/digestion/absorption of nutrients

Participant states he/she has an eating disorder, severe dementia or delirium, history of significant neurological or psychiatric disorder, alcoholism, substance abuse, or other conditions that may interfere with compliance with study protocol procedures in the opinion of the PI or study physician

Participant states that he/she is a participant in a concomitant trial or trial of a non-registered drug (or is within the 30 day follow-up period for such a trial)

---

[1] American College of Sports Medicine. *ACSM's Guidelines for Exercise Testing and Prescription.* 10th ed. Wolters Kluwer Publishers; 2017.

[2] Cruz-Jentoft AJ, Baeyens JP, Bauer JM, Boirie Y, Cederholm T, Landi F *et al.* Sarcopenia: European consensus on definition and diagnosis: Report of the European Working Group on

Sarcopenia in Older People. *Age Ageing* 2010;**39**:412–423
