# Supplementary material for: Differences in muscle energy metabolism and metabolic flexibility between sarcopenic and nonsarcopenic older adults
Source: J Cachexia Sarcopenia Muscle. 2022 Feb 17;13(2):1224–37. doi: 10.1002/jcsm.12932 (PMC8978004; doi:10.1002/jcsm.12932)
Supplement: Supplementary file 8 — Table S2. Strength and function assessments performed by participants in the Screening Visit to determine eligibility and classification of sarcopenic status. [file JCSM-13-1224-s007.pdf]

Differences in Muscle Energy Metabolism and Metabolic Flexibility between Sarcopenic and Non-sarcopenic Older Adults, Journal of Cachexia, Sarcopenia and Muscle.

Marni E. Shoemaker, Suzette L. Pereira, Vikkie A. Mustad, Zachary M. Gillen, Brianna D. McKay, Jose M. Lopez-Pedrosa, Ricardo Rueda, Joel T. Cramer*

* College of Health Sciences, The University of Texas at El Paso, El Paso, TX 79968, USA, jtcramer@utep.edu

Supplementary Table S2. Strength and function assessments performed by participants in the Screening Visit to determine eligibility and classification of sarcopenic status.

Handgrip Strength	
	Handgrip strength was measured with a handheld dynamometer that was standardized and adjusted to the second joint of the finger just below the handle. Participants adducted the right arm to their side with 90° flexion at the elbow and squeezed the dynamometer handle as forcefully as possible for three to five seconds. Three trials were performed, with the average of the trials used as the final strength value.
Short Physical Performance Battery	
Balance	The balance component tested the participant's balance at three progressively harder values. A score was provided based on completion or time to failure.
Gait Speed	Two trials were timed over four meters at normal walking speed. If the second gait speed trial was more than 10% different than the first trial, a third trial was conducted.
Chair Stand	Participants were seated on a chair with their feet planted on the ground and arms crossed on their chest. Participants were timed to see how long it

took to rise from the chair and return to the seated position five times.
