# Supplementary material for: Differences in muscle energy metabolism and metabolic flexibility between sarcopenic and nonsarcopenic older adults
Source: J Cachexia Sarcopenia Muscle. 2022 Feb 17;13(2):1224–37. doi: 10.1002/jcsm.12932 (PMC8978004; doi:10.1002/jcsm.12932)
Supplement: Supplementary file 9 — Table S5. Means ± standard deviations (SD) of energy intake and macronutrient composition of food consumed the three days prior to the experimental visit. P‐values are type I errors of the interactions and main effects. [file JCSM-13-1224-s009.pdf]

Differences in Muscle Energy Metabolism and Metabolic Flexibility between Sarcopenic and Non-sarcopenic Older Adults, Journal of Cachexia, Sarcopenia and Muscle.

Marni E. Shoemaker, Suzette L. Pereira, Vikkie A. Mustad, Zachary M. Gillen, Brianna D. McKay, Jose M. Lopez-Pedrosa, Ricardo Rueda, Joel T. Cramer*

* College of Health Sciences, The University of Texas at El Paso, El Paso, TX 79968, USA, jtcramer@utep.edu

Supplementary Table S5. Means ± standard deviations (SD) of energy intake and macronutrient composition of food consumed the three days prior to the experimental visit. P-values are type I errors of the interactions and main effects.

			NON-SARCOPENIC						SARCOPENIC						Interaction Effect (Sarcopenia x Sex)	Main Effect (Sarcopenia)	Main Effect (Sex)
			Males			Females			Males			Females					
	Sample Size	(n)	5			6			6			5					
Day 1	Carbohydrate	(g)	253.0	±	116.8	265	±	111.6	243.7	±	74.9	241.0	±	82.2	p = 0.864	p = 0.697	p = 0.913
	Fat	(g)	72.0	±	34.5	92.7	±	42.3	91.2	±	43.7	64.8	±	39.9	p = 0.193	p = 0.805	p = 0.872
	Protein	(g)	89.0	±	24.7	77.5	±	30.6	96.5	±	40.9	79.6	±	44.7	p = 0.863	p = 0.760	p =0 .371
	Energy Intake	(kcals)	2081.4	±	431.4	2306.0	±	550.8	2193.3	±	773.9	1843.8	±	813.0	p = 0.325	p = 0.545	p = 0.828
Day 2	Carbohydrate	(g)	303.4	±	138.5	243.0	±	84.9	245.5	±	118.7	242.4	±	83.7	p = 0.545	p = 0.536	p = 0.502
	Fat	(g)	81.2	±	53.3	53.3	±	18.9	85.0	±	29.8	71.6	±	30.8	p = 0.504	p = 0.312	p = 0.068
	Protein	(g)	83.4	±	21.8	58.5	±	23.6	83.3	±	30.2	87.0	±	36.2	p = 0.255	p = 0.257	p = 0.394
	Energy Intake	(kcals)	2418.2	±	590.8	1711.2	±	551.4	2141.0	±	710.7	1939.4	±	653.9	p = 0.361	p = 0.929	p = 0.110
Day 3	Carbohydrate	(g)	325.2	±	112.7	215.5	±	106.2	328.5	±	154.8	224.0	±	66.3	p = 0.959	p = 0.907	**p = 0.046**
	Fat	(g)	114.6	±	51.8	111.0	±	76.3	81.3	±	36.2	66.0	±	13.1	p = 0.792	p = 0.091	p = 0.671
	Protein	(g)	100.0	±	34.6	70.5	±	26.3	81.7	±	25.1	67.2	±	21.7	p = 0.526	p = 0.365	p = 0.075
	Energy Intake	(kcals)	2669.6	±	880.6	2006.5	±	598.5	1731.8	±	398.4	1731.8	±	398.4	p = 0.992	p = 0.398	p = 0.055
