# Supplementary material for: Differences in muscle energy metabolism and metabolic flexibility between sarcopenic and nonsarcopenic older adults
Source: J Cachexia Sarcopenia Muscle. 2022 Feb 17;13(2):1224–37. doi: 10.1002/jcsm.12932 (PMC8978004; doi:10.1002/jcsm.12932)
Supplement: Supplementary file 10 — Table S8. Respiratory Quotient (RQ) and substrate utilization at baseline and 180 minutes post‐prandial a CHO‐rich meal. Values are means ± standard deviations (SD). P‐values are type I errors of independent t‐tests. [file JCSM-13-1224-s003.pdf]

Differences in Muscle Energy Metabolism and Metabolic Flexibility between Sarcopenic and Non-sarcopenic Older Adults, Journal of Cachexia, Sarcopenia and Muscle.

Marni E. Shoemaker, Suzette L. Pereira, Vikkie A. Mustad, Zachary M. Gillen, Brianna D. McKay, Jose M. Lopez-Pedrosa, Ricardo Rueda, Joel T. Cramer[*]

[*] College of Health Sciences, The University of Texas at El Paso, El Paso, TX 79968, USA, jtcramer@utep.edu

Supplementary Table S8. Respiratory Quotient (RQ) and substrate utilization at baseline and 180 minutes post-prandial a CHO-rich meal. Values are means ± standard deviations (SD). P-values are type I errors of independent t-tests.

	**Non-Sarcopenic**	**Sarcopenic**	
**Time**	Mean ± SD	Mean ± SD	*p value*
**Respiratory Quotient**			
**0**	0.72 ± 0.04	0.76 ± 0.04	**p=0.025**
**15**	0.84 ± 0.05	0.89 ± 0.05	p=0.086 [⁂]
**30**	0.84 ± 0.05	0.89 ± 0.06	p=0.143 [⁂]
**45**	0.83 ± 0.04	0.86 ± 0.05	p=0.538 [⁂]
**60**	0.81 ± 0.03	0.83 ± 0.04	p=0.976 [⁂]
**75**	0.78 ± 0.04	0.82 ± 0.05	p=0.433 [⁂, #]
**90**	0.76 ± 0.04	0.81 ± 0.03	p=0.053 [⁂, #, §, †, ‡]
**120**	0.77 ± 0.04	0.82 ± 0.03	**p=0.007** [⁂, #, §, †, ‡]
**150**	0.76 ± 0.03	0.80 ± 0.04	**p=0.010** [⁂, #, §, †, ‡]

**180**	0.74 ± 0.04	0.80 ± 0.04	**p=0.002** [⁂, #, §, †, ‡]
**CHO oxidation normalized to FFM (g·min^{-1}·kg^{-1})**			
**0**	0.0004 ± 0.007	0.001 ± 0.001	***0.047***
**15**	0.004 ± 0.001	0.004 ± 0.001	*0.422* [*, ⁂]
**30**	0.004 ± 0.002	0.004 ± 0.002	*0.502* [⁂]
**45**	0.004 ± 0.001	0.003 ± 0.001	*0.741* [*, ⁂]
**60**	0.003 ± 0.001	0.003 ± 0.001	*0.274* [⁂]
**75**	0.003 ± 0.001	0.003 ± 0.001	*0.851* [*, ⁂, #, †]
**90**	0.002 ± 0.001	0.002 ± 0.001	*0.423* [⁂, #, §, †, ‡]
**120**	0.002 ± 0.001	0.002 ± 0.001	***0.020*** [⁂, #, §, †, ‡]
**150**	0.001 ± 0.001	0.002 ± 0.001	*0.051* [⁂, #, §, †, ‡, ‖]
**180**	0.001 ± 0.001	0.002 ± 0.001	***0.010*** [#, §, †, ‡, ‖]
**Fat oxidation normalized to FFM (g·min^{-1}·kg^{-1})**			
**0**	0.002 ± 0.000	0.002 ± 0.001	***0.020***
**15**	0.001 ± 0.000	0.001 ± 0.001	***0.038*** [⁂]
**30**	0.001 ± 0.000	0.001 ± 0.001	*0.061* [⁂]
**45**	0.001 ± 0.000	0.001 ± 0.001	*0.176* [⁂]
**60**	0.002 ± 0.000	0.001 ± 0.000	*0.334* [⁂]
**75**	0.002 ± 0.000	0.001 ± 0.000	*0.100* [⁂, #]
**90**	0.002 ± 0.000	0.002 ± 0.000	***0.009*** [⁂. #, §, †]
**120**	0.002 ± 0.000	0.001 ± 0.000	***0.009*** [#, §, †]
**150**	0.002 ± 0.000	0.002 ± 0.000	***0.004*** [#, §, †, ‡]
**180**	0.002 ± 0.000	0.002 ± 0.000	***0.004*** [#, §, †, ‡]

p-values in bold indicate differences between non-sarcopenic (NS) and sarcopenic (S) groups from planned comparisons using independent samples t-tests. * indicates a significant difference between males and females. ⁂ indicates a significant difference from baseline. # indicates a significant difference from 15 min. §indicates a significant difference from 30 min. † indicates a significant difference from 45 min. ‡ indicates a significant difference from 60 min. ‖ indicates a significant difference from 75 min. ¶ indicates a significant difference from 90 min. Ø indicates a significant difference from 120 min. $ indicates a significant difference from 150 min ($p \leq 0.05$).
