# Supplementary material for: Differences in muscle energy metabolism and metabolic flexibility between sarcopenic and nonsarcopenic older adults
Source: J Cachexia Sarcopenia Muscle. 2022 Feb 17;13(2):1224–37. doi: 10.1002/jcsm.12932 (PMC8978004; doi:10.1002/jcsm.12932)
Supplement: Supplementary file 11 — Table S9. Venous blood samples taken at baseline and 180 minutes postprandial a CHO‐rich meal. Values are means ± standard deviations (SD). P‐values are type I errors of independent t‐tests. [file JCSM-13-1224-s012.pdf]

Differences in Muscle Energy Metabolism and Metabolic Flexibility between Sarcopenic and Non-sarcopenic Older Adults, Journal of Cachexia, Sarcopenia and Muscle.

Marni E. Shoemaker, Suzette L. Pereira, Vikkie A. Mustad, Zachary M. Gillen, Brianna D. McKay, Jose M. Lopez-Pedrosa, Ricardo Rueda, Joel T. Cramer*

* College of Health Sciences, The University of Texas at El Paso, El Paso, TX 79968, USA, jtcramer@utep.edu

Supplementary Table S9. Venous blood samples taken at baseline and 180 minutes post-prandial a CHO-rich meal. Values are means ± standard deviations (SD). P-values are type I errors of independent t-tests.

	**Non-Sarcopenic**	**Sarcopenic**	
**Glucose (mg·dL^{-1})**			
**Time**	Mean ± SD	Mean ± SD	*p value*
**0**	107.70 ± 8.43	144.64 ± 43.22	***0.018***
**15**	132.70 ± 39.45	164.45 ± 68.93	*0.217*
**30**	152.67 ± 24.36	172.44 ± 89.58	*0.532*
**45**	140.89 ± 29.61	197.00 ± 81.97	*0.071*
**60**	134.20 ± 37.64	202.78 ± 90.42	***0.042***
**75**	125.30 ± 35.71	186.11 ± 48.10	***0.006*** ‖
**90**	116.30 ± 46.18	156.89 ± 47.37	*0.076*
**120**	112.70 ± 44.14	126.88 ± 38.16	*0.483*
**150**	113.90 ± 46.90	150.00 ± 48.01	*0.128*

**180**	113.30 ± 47.52	135.38 ± 21.88	*0.244*
**Insulin (mU·L^{-1})**			
**0**	3.38 ± 1.96	3.40 ± 1.04	*0.978*
**15**	7.76 ± 4.91	5.45 ± 6.91	*0.304* ⁂
**30**	22.31 ± 15.80	17.03 ± 9.87	*0.407* ⁂, #
**45**	26.85 ± 16.86	20.44 ± 12.58	*0.375* ⁂, #
**60**	22.28 ± 14.67	18.48 ± 12.25	*0.551* ⁂, #
**75**	23.10 ± 15.49	15.63 ± 7.58	*0.198* ⁂, #
**90**	18.75 ± 10.78	17.46 ± 9.82	*0.790* ⁂
**120**	9.31 ± 5.67	10.42 ± 5.20	*0.663* ⁂, †, ‡, ‖, ¶
**150**	5.45 ± 3.31	9.27 ± 5.10	*0.068* §, †, ‡, ‖, ¶
**180**	2.70 ± 2.14	3.87 ± 2.87	*0.322* §, †, ‡, ‖, ¶, ø, $

p-values in bold indicate differences between non-sarcopenic (NS) and sarcopenic (S) groups from planned comparisons using independent samples t-tests. * indicates a significant difference between males and females. ⁂ indicates a significant difference from baseline. # indicates a significant difference from 15 min. § indicates a significant difference from 30 min. † indicates a significant difference from 45 min. ‡ indicates a significant difference from 60 min. ‖ indicates a significant difference from 75 min. ¶ indicates a significant difference from 90 min. Ø indicates a significant difference from 120 min. $ indicates a significant difference from 150 min ($p \leq 0.05$).
