# Supplementary material for: Differences in muscle energy metabolism and metabolic flexibility between sarcopenic and nonsarcopenic older adults
Source: J Cachexia Sarcopenia Muscle. 2022 Feb 17;13(2):1224–37. doi: 10.1002/jcsm.12932 (PMC8978004; doi:10.1002/jcsm.12932)
Supplement: Supplementary file 12 — Table S10. Substrate utilization during a submaximal aerobic test at 50–60% of estimated V̇O2. Values are means ± standard deviations (SD). P‐values are type I errors of independent t‐tests. [file JCSM-13-1224-s008.pdf]

Differences in Muscle Energy Metabolism and Metabolic Flexibility between Sarcopenic and Non-sarcopenic Older Adults, Journal of Cachexia, Sarcopenia and Muscle.

Marni E. Shoemaker, Suzette L. Pereira, Vikkie A. Mustad, Zachary M. Gillen, Brianna D. McKay, Jose M. Lopez-Pedrosa, Ricardo Rueda, Joel T. Cramer*

* College of Health Sciences, The University of Texas at El Paso, El Paso, TX 79968, USA, jtcramer@utep.edu

Supplementary Table S10. Substrate utilization during a submaximal aerobic test at 50 - 60% of estimated V̇O2. Values are means ± standard deviations (SD). P-values are type I errors of independent t-tests.

	**Non-Sarcopenic**	**Sarcopenic**	
**Respiratory Quotient (RQ)**			
**Time**	Mean ± SD	Mean ± SD	*p-value*
**0 – 2**	0.83 ± 0.04	0.85 ± 0.05	*0.312*
**2 – 4**	0.85 ± 0.04	0.88 ± 0.06	*0.182* ⁂
**4 – 6**	0.85 ± 0.03	0.89 ± 0.06	*0.066* ⁂
**6 – 8**	0.86 ± 0.03	0.90 ± 0.06	***0.039*** ⁂
**8 – 10**	0.86 ± 0.03	0.90 ± 0.06	*0.095* ⁂
**CHO Oxidation normalized to FFM ($g \cdot min^{-1} \cdot kg^{-1}$)**			
**0 – 2**	0.011 ± 0.005	0.012 ± 0.004	*0.621*
**2 – 4**	0.013 ± 0.004	0.015 ± 0.004	*0.544* ⁂
**4 – 6**	0.014 ± 0.004	0.016 ± 0.004	*0.533* ⁂

**6 – 8**	0.015 ± 0.004	0.016 ± 0.005	*0.502* ⁂
**8 – 10**	0.015 ± 0.004	0.017 ± 0.005	*0.435* ⁂
**Fat Oxidation normalized to FFM ($g \cdot min^{-1} \cdot kg^{-1}$)**			
**0 – 2**	0.006 ± 0.001	0.005 ± 0.002	*0.280*
**2 – 4**	0.005 ± 0.002	0.004 ± 0.002	*0.127*
**4 – 6**	0.005 ± 0.012	0.004 ± 0.002	*0.053* ⁂
**6 – 8**	0.005 ± 0.001	0.003 ± 0.002	***0.033*** ⁂
**8 – 10**	0.005 ± 0.001	0.003 ± 0.002	*0.087* ⁂

p-values in bold indicate differences between non-sarcopenic (NS) and sarcopenic (S) groups from planned comparisons using independent samples t-tests. * indicates a significant difference between males and females. ⁂ indicates a significant difference from 0 – 2 min. ($p \leq 0.05$).
