# Supplementary material for: Differences in muscle energy metabolism and metabolic flexibility between sarcopenic and nonsarcopenic older adults
Source: J Cachexia Sarcopenia Muscle. 2022 Feb 17;13(2):1224–37. doi: 10.1002/jcsm.12932 (PMC8978004; doi:10.1002/jcsm.12932)
Supplement: Supplementary file 13 — Table S11. Substrate utilization during an anaerobic fatiguing test at 30% of estimated one‐repetition maximum. P‐values are type I errors of independent t‐tests. [file JCSM-13-1224-s006.pdf]

Differences in Muscle Energy Metabolism and Metabolic Flexibility between Sarcopenic and Non-sarcopenic Older Adults, Journal of Cachexia, Sarcopenia and Muscle.

Marni E. Shoemaker, Suzette L. Pereira, Vikkie A. Mustad, Zachary M. Gillen, Brianna D. McKay, Jose M. Lopez-Pedrosa, Ricardo Rueda, Joel T. Cramer*

* College of Health Sciences, The University of Texas at El Paso, El Paso, TX 79968, USA, jtcramer@utep.edu

Supplementary Table S11. Substrate utilization during an anaerobic fatiguing test at 30% of estimated one-repetition maximum. P-values are type I errors of independent t-tests.

	**Non-Sarcopenic**	**Sarcopenic**	
**Respiratory Quotient (RQ)**			
**Effort**	Mean ± SD	Mean ± SD	*p-value*
**0 – 20%**	0.91 ± 0.06	0.91 ± 0.06	*0.767*
**20 – 40%**	0.91 ± 0.08	0.91 ± 0.06	*0.862*
**40 – 60%**	0.91 ± 0.8	0.91 ± 0.07	*0.959*
**60 – 80%**	0.94 ± 0.05	0.92 ± 0.07	*0.478*
**80 – 100%**	0.98 ± 0.07	0.93 ± 0.06	*0.055*
**CHO Oxidation normalized to FFM (g·min^{-1}·kg^{-1})**			
**0 – 20%**	0.004 ± 0.001	0.004 ± 0.002	*0.907*
**20 – 40%**	0.005 ± 0.002	0.006 ± 0.003	*0.989* ⁂
**40 – 60%**	0.007 ± 0.003	0.007 ± 0.003	*0.753* ⁂, #

**60 – 80%**	0.011 ± 0.002	0.007 ± 0.003	***0.015*** ⁂, #, §
**80 – 100%**	0.013 ± 0.008	0.008 ± 0.004	*0.097* ⁂, #
**Fat Oxidation normalized to FFM (g·min^{-1}·kg^{-1})**			
**0 – 20%**	0.0009 ± 0.0009	0.0008 ± 0.0008	*0.696*
**20 – 40%**	0.0010 ± 0.0010	0.0009 ± 0.0008	*0.830*
**40 – 60%**	0.0015 ± 0.0016	0.0015 ± 0.0016	*0.461*
**60 – 80%**	0.0012 ± 0.0012	0.0012 ± 0.0012	*0.541*
**80 – 100%**	0.0003 ± 0.0014	0.0010 ± 0.0009	*0.227*

p-values in bold indicate differences between non-sarcopenic (NS) and sarcopenic (S) groups from planned comparisons using independent samples t-tests. * indicates a significant difference between males and females. ⁂ indicates a significant difference from 0 – 20%. # indicates a significant difference from 20 – 40%. § indicates a significant difference from 40 – 60%. ($p \leq 0.05$).
